# High-Resolution Doppler and Azimuth Estimation and Target Detection in HFSWR: Experimental Study

**DOI:** 10.3390/s22093558

**Published:** 2022-05-07

**Authors:** Dragan Golubović, Miljko Erić, Nenad Vukmirović

**Affiliations:** 1 University of Belgrade, School of Electrical Engineering, 11120 Belgrade, Serbia; miljko.eric@vlatacom.com (M.E.); nenad.vukmirovic@ic.etf.bg.ac.rs (N.V.); 2 Vlatacom Institute, 11070 Belgrade, Serbia; 3 University of Belgrade, Innovation Center of the School of Electrical Engineering, 11120 Belgrade, Serbia

**Keywords:** HFSWR, OTHR, high-resolution methods, Range–Doppler map, ship detection, WERA

## Abstract

In this paper, we present a new high-resolution algorithm for primary signal processing in High Frequency Surface Wave Radar (HFSWR). The algorithm has been developed to achieve and improve primary signal processing performance in existing HFSWR radars in terms of radar target detection. The proposed algorithm is based on a high-resolution estimate of the Range–Doppler (RD-HR) map using given number of frames in the selected integration period. RD-HR maps are formed at every antenna in receive antenna array. Target detection is based on an RD-HR map averaged across all the antennas. Azimuth estimation is performed by a high-resolution MUSIC-type algorithm that is executed for all detections we found in the RD-HR map. The existence of strong Bragg’s lines in the RD-HR map complicates the detection process but the contrast of the RD-HR map as well as the detectability of targets on the RD-HR map is significantly better compared to the RD-FFT map used by many existing radars, such as WERA.

## 1. Introduction

Recently, in many countries which have direct access to the sea, great importance has been attached to the monitoring of the Exclusive Economic Zone (EEZ), which is defined in accordance with the United Nations Convention on the Law of the Sea. This is very important because many illegal activities can be carried out beyond the horizon, which are under the jurisdiction of the EEZ of a particular country. In this regard, High Frequency Surface Wave Radars (HFSWR) are widely used in the past for maritime surveillance of ships at ranges up to 200 nautical miles [1,2,3].

Various theoretical as well as practical implementation aspects of HFSWR radars are highlighted in numerous published works [4,5,6,7,8,9,10,11,12].

Despite the fact that the principles of HFSWR radars are theoretically well studied and clarified, and practically implemented and verified [13,14,15,16], HFSWR radars are still the subject of intensive research and development with the aim to improve detectability and resolution performance of targets with small radar cross-section (small boats, UAV, drones, etc.). The motivation for those research and development efforts are related to the application of HFSWR radars for monitoring of illegal crime activities, drug trafficking, attack on petrol platforms and strategic objects, etc. Therefore, the focus of this paper is to develop and propose new high resolution algorithm and new detection scheme in order to improve detection and resolution performance of HFSWR radars based on FMCW principle.

We have shown here that multidimensional signal at the output of dechirper of FMCW HFSWR radar (with neglected coupling between domains) can be modeled as superposition of ionospheric interference, sea clutter, additive noise and attenuated sinusoids (cissoids) in 3D space (fast time domain, slow time domain and spatial domain), each of them correspond to the range, Doppler/radial velocity and azimuth of one target in multi-target scenario, typical for HFSWR radars. Therefore, the task of signal processing in FMCW HFSWR radar is to solve detection/estimation problem:

- to detect the number of superposed cisoids, what is equivalent to the detection targets number in multi-target scenario;
- to estimate frequencies of those cisoids in fast time, slow time and spatial domain, what is equivalent to the estimation of their parameters (range, Doppler/radial velocity and azimuth).

If the receiving antenna array is linear and uniform, the estimation problem is usually solved by 3D Fourier transform. Such solution is applied in many practical implementations of FMCW HFSWR radars. As a result, so called range/Doppler/azimuth (RDA map) is provided. It is usually followed by CFAR detection of targets in RDA map. That is conventional approach of the signal processing in FMCW HFSWR radar.

It is well known that the target detection performance of HFSWR radars are basically limited by the presence of sea clutter, ionospheric and external interference and additive noise. Two main groups for target detection methods in FMCW HFSWR radars are proposed so far: Constant False Alarm Ratio (CFAR) based methods and image processing based methods. CFAR detection methods with its various variants are usually used in FMCW HFSWR radars [17,18,19,20]. However, some other detection methods like [21,22,23,24,25,26] are proposed. In this paper, we proposed new detection algorithm based on [27] which is primarily used in Image Processing, not in HFSWR.

The key problem of detection process is how to mitigate external interference. Various schemes for interference mitigation as a preprocessing step are proposed so far. In regard of this, target detection is still a challenging research problem, especially in the context of the application of high-resolution methods for range/Doppler/azimuth estimation, as presented in [28,29,30,31,32,33,34,35,36,37,38,39,40,41,42,43,44,45].

If RDA map is estimated by classical 3D FFT, resolution in Range, Doppler/Radial velocity and Azimuth domain are limited by system parameters such as chirp signal bandwidth (range), the period of integration (Doppler/radial velocity) and the number of antennas in the receiving antenna arrays. Increasing of these parameters leads to better detectability and better resolution properties of radar in each domain. Increasing the integration period improves the resolution of the Doppler estimate, but this increase is limited by the Coherent integration time related to the physics of wave propagation. The integration period must be shorter than the Coherent integration time. On the other hand, it is implicitly assumed that the target within the integration period does not change the radial velocity, which is not a valid assumption for large integration periods. Increasing the number of antennas in the antenna array improves the resolution properties, but this results in an increase in the physical space for placing the antenna array.

The RD-HR map is the key to the high-resolution method proposed. In that case, criterion functions are calculated on all antennas for a set of discrete values of normalized Doppler frequencies, for the range of Doppler frequencies of interest, and with a grid resolution that is many times better than the FFT Doppler resolution, thus obtaining a zoomed high-resolution RD-HR map. The high-resolution properties of the obtained RD-HR maps and better detectability and contrast of targets in the RD-HR map in relation to the RD-FFT map are clearly observed in rest of the paper. MUSIC-type high-resolution algorithm is used to form the covariance matrix that is the basis for the application of most high-resolution methods.

The main contributions of this paper are as follows. The high-resolution method for estimating the RD-HR map (uniform and more computationally efficient non-uniform variant) is proposed. Then the Doppler shift was compensated before high-resolution azimuth estimation, which is also newly proposed. The contribution is also in a novel detection algorithm that comes from the field of Image processing whose kernel function is more convenient to the morphologies of peaks of criterion functions of high-resolution methods than the classic CFAR, which is more suitable for use in 3D FFT RDA map estimation.

The rest of this paper is organized as follows. Section 2 introduces the system and signal model used to generate results and to test the proposed method. In Section 3, we presented detailed algorithm description. Here, we explained 2 variants of the algorithm: High-resolution Range–Doppler map estimation (both for uniform and non-uniform in slow time domain). We explained also the detection of targets on the Range–Doppler map, and a novel method for azimuth detection with improved accuracy and numerical complexity. We discuss some of the experimental results in Section 4 and provide concluding remarks in Section 5. The derivation of dechirped signal was presented in the Appendix A.

## 2. System and Signal Model

### 2.1. System Model

**Figure 1:**
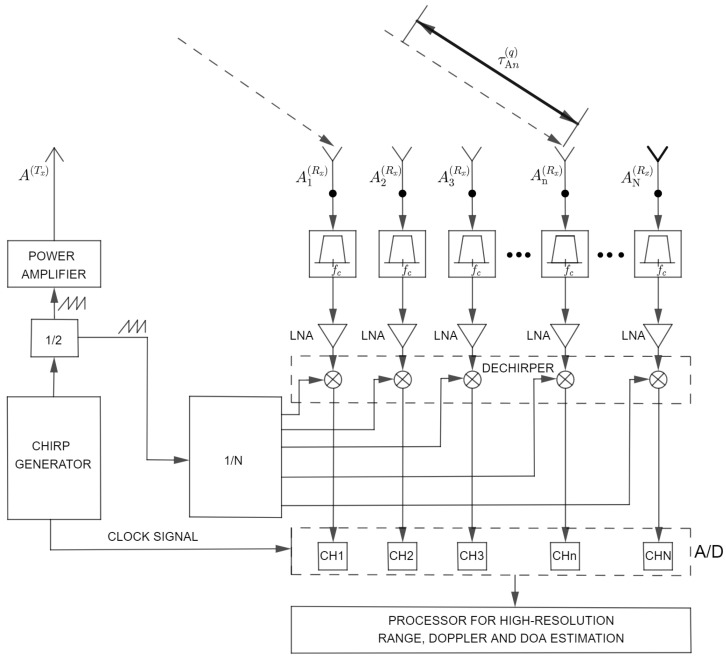
System model.

The High Frequency Surface Wave radar (HFSW) to be analyzed in this paper uses Frequency Modulated Continuous Waves (FMCW) which are vertically polarized. Specific propagation of waves is the key for over-the horizon-coverage. This is the most important difference between HF radar and standard microwave surveillance radars. They operate in HF spectrum 3–30 MHz. Exact frequency selection depends on specific requirements. A typical configuration is given in Figure 1.

Generally, the radar consists of 3 subsystems: transmitter (Tx) antenna array, receiver (Rx) antenna array and transceiver that can be in one place or physically separated. Positions of Tx antennas are known. One of the antennas in the Tx antenna array is marked with $A^{\left(\mathrm{Tx}\right)}$. The transmitter should provide a signal at a specific frequency and a sufficient power level to allow detection of targets at great distances. The output amplifier should provide a variable level of RF signal to optimize the system in different operating conditions and environmental influences. The following requirements are set when designing the Tx antenna system: the radiated energy should be maximally directed in the direction of the sea and the system must radiate as little as possible towards the Rx antenna array. A problem in FMCW systems is the isolation between the Tx and Rx antenna array. The signal level of the direct wave from the Tx antenna to the Rx antennas must not saturate the Rx.

The Rx antenna array is linear, but other geometries of antenna arrays can be used. Antennas are installed parallel to the sea shore. The center of the Tx array should be in line with the Rx antenna array. Positions of Rx antennas are known, too. They are connected to *N* collocated receiving channels by calibrated cables (coaxial or fiber optic). The *n*-th antenna in the Rx antenna array is marked with $A_{n}^{\left(\mathrm{Rx}\right)}$.

The Rx must provide mutual coherence for all receiving channels (one channel for each antenna). In the Rx, instead of performing classical demodulation, the received signals are multiplied by a conjugated replica of the Tx waveform (generated in Chirp Generator) in a dechirper. Finally, the dechirped signals are sampled and sent to the block for signal processing.

HFSWR radar WERA located on Ibeju Lekki, Nigeria, was used as a reference system for signal acquisition and performance comparison. On this specific test site, an Rx antenna array consists of 16 monopole antennas, where the distance between antennas in the antenna array is $0.45\lambda_{c}$ ($\lambda_{c}$ is the wavelength of the used carrier frequency) and Rx antenna array aperture is $6.75\lambda_{c}$. Also, the Tx planar array consisting of four antennas is used (two active and two passive antennas as reflectors). Since it is a continuous radar, the critical parameter of such a system is the isolation between Tx and Rx, which is systematically provided by careful design of Tx and Rx antenna array geometry and careful receiver design. Due to strong Tx-Rx wave, the reduction of the dynamic range of the analog signal at the input to the A/D converter is needed. In practical implementations, Rx and Tx arrays have to be separated as much as possible. The selected distance is limited by the size of the site on which the radar is placed. In the specific installation, the distance between the Tx and Rx antenna arrays is 1200 m. This length is sufficient for all frequencies of interest, and this is taken into account when designing the site.

Another solution to improve the isolation between Tx and Rx is to install a notch filter at the dechirper output which suppresses signals around 0 Hz (DC), in order to reduce the impact of transmitter leakage. It is important to note that this filter does not influence the stationary targets located away from the radar, but only close targets. The bandwidth of the transmitted signal is 100 kHz and the chirp duration is 0.260022 s.

The Tx signal is amplified to the desired level using a power amplifier and fed to the transmit antenna array. In existing HFSWRs, it is common to use power levels 500–1000 W. At the test site, we used a power level of 500 W. From each of the 16 Rx antennas, the signal is first filtered in order to suppress out-of-band components, then it is amplified to the level needed for A/D conversion and further processing.

### 2.2. Signal Model

**Figure 2:**
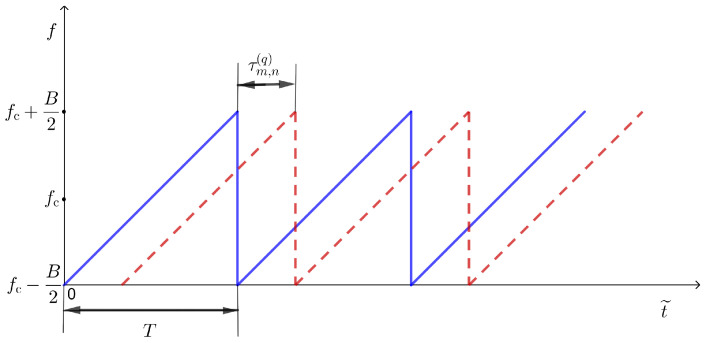
Transmitted chirp signal (solid blue line) and received chirp signal (dashed red line).

The Tx and the Rx array are synchronized in time and phase. The Tx transmits a periodic sequence of chirps at a carrier frequency $f_{c}$. In Figure 2 the proposed signal model was shown.

Continuous time variable $\tilde{t}$ spans the entire time-axis, whereas *t* is the time elapsed from the beginning of a chirp.

(1) $$-\infty <\tilde{t}<+\infty$$

(2) $$t\equiv \tilde{t}\;\left(\mathrm{mod}\;T\right),\;t\in \left[0,T\right)$$

For convenience, besides the continuous time variable $\tilde{t}$, we also define

(3) $$\begin{array}{cc} m & =\left\lfloor \frac{\tilde{t}}{T}\right\rfloor \end{array}$$

(4) $$\begin{array}{cc} \tilde{t} & =t+mT,\;m\in Z, \end{array}$$

where *m* is the index of a chirp (also called “slow time”) and *t* is the time variable within a chirp (also called “fast time”). Note that $\tilde{t}$, *t*, and *m* are mutually dependent variables and that $\tilde{t}$ can be equivalently expressed by the pair $\left(m,t\right)$.

A single chirp, denoted by c(t), is modeled as

(5) $$c\left(t\right)=e^{j2\pi \left(\left(f_{c}-\frac{B}{2}\right)t+\frac{B}{2T}t^{2}\right)}.$$

The transmitted signal, denoted by $r\left(\tilde{t}\right)$, is a periodic sequence of chirps with period *T*, defined as

(6) $$\begin{array}{cc} r\left(t\right)=c\left(t\right), & \;0\leq t<T \end{array}$$

(7) $$\begin{array}{cc} r\left(\tilde{t}\right)=r\left(\tilde{t}+T\right), & \;-\infty <\tilde{t}<+\infty . \end{array}$$

Time delay of the transmitted chirp signal of the *q*-th target on the range $R^{\left(q\right)}$ on the *n*-th antenna is modeled as

(8) $$\tau_{n}^{\left(q\right)}\left(\tilde{t}\right)=\frac{2R_{m}^{\left(q\right)}}{c}+\frac{2v_{m}^{\left(q\right)}}{c}t+\tau_{An}^{\left(q\right)},$$

where *c* is the wave propagation velocity, $\tau_{An}^{\left(q\right)}$ is the relative delay at the *n*-th receive antenna w.r.t. the referent point of the receiving array, $R_{m}^{\left(q\right)}$ is the range to the target relative to the referent point of the Rx array and $v_{m}^{\left(q\right)}$ is the radial velocity of the target during the *m*-th chirp (and is assumed constant during each chirp).

Signal model of the received signal reflected from the *q*-th target on *n*-th antenna is modeled as

(9) $$x_{n}^{\left(q\right)}\left(\tilde{t}\right)=\left\{\begin{array}{cc} a^{\left(q\right)}r\left(t-\tau_{n}^{\left(q\right)}\left(\tilde{t}\right)\right), & \mathrm{for}\;\tau_{n}^{\left(q\right)}\left(\tilde{t}\right)<t<T\\ a^{\left(q\right)}r\left(t-\tau_{n}^{\left(q\right)}\left(\tilde{t}\right)+T\right), & \mathrm{for}\;0<t<\tau_{n}^{\left(q\right)}\left(\tilde{t}\right) \end{array}\right.$$

where τ is the two-way propagation time for the target and a∈R is an attenuation factor. The model is continous in fast time, and discrete in slow time and antenna domain.

The signal at the output of the dechirper is given by

(10) $$y_{n}^{\left(q\right)}\left(\tilde{t}\right)=x_{n}^{\left(q\right)}\left(\tilde{t}\right)r{\left(\tilde{t}\right)}^{*}=\left\{\begin{array}{cc} a^{\left(q\right)}r\left(t-\tau_{n}^{\left(q\right)}\left(\tilde{t}\right)\right)r{\left(\tilde{t}\right)}^{*}, & \mathrm{for}\;\tau_{n}^{\left(q\right)}\left(\tilde{t}\right)<t<T\\ a^{\left(q\right)}r\left(t-\tau_{n}^{\left(q\right)}\left(\tilde{t}\right)+T\right)r{\left(\tilde{t}\right)}^{*}, & \mathrm{for}\;0<t<\tau_{n}^{\left(q\right)}\left(\tilde{t}\right) \end{array}\right.$$

The complete derivation of the equations is given in the Appendix A, whereas the approximated equations, under some assumptions, are given in the Appendix B. So, we have an approximated model of the dechirped signal, as shown in the following 2 equations.

In the first case, when $\tau_{m,n}^{\left(q\right)}<t<T$, the dechirped signal is modeled as

(11) $$\begin{array}{cc} y_{n}^{\left(q\right)}\left(\tilde{t}\right) & =a^{\left(q\right)}e^{j2\pi R_{0}^{\left(q\right)}\left(-\frac{2tB}{cT}-\frac{2f_{c}-B}{c}\right)}\times e^{j2\pi v^{\left(q\right)}\left(-\frac{2f_{c}-B}{c}mT-\frac{2mtB}{c}\right)}\\ \; & \times e^{j2\pi \tau_{An}^{\left(q\right)}\left(\frac{-Bt}{T}-f_{c}+\frac{B}{2}\right)}\times e^{j2\pi \tau_{An}^{\left(q\right)}R_{0}^{\left(q\right)}\frac{2B}{cT}}\\ \; & \times e^{j2\pi \tau_{An}^{\left(q\right)}v^{\left(q\right)}\frac{2Bm}{c}}\times e^{j2\pi {\left(\tau_{An}^{\left(q\right)}\right)}^{2}\frac{B}{2T}}. \end{array}$$

In the second case, when $0<t<\tau_{m,n}^{\left(q\right)}$, the dechirped signal is modeled as:

(12) $$\begin{array}{cc} y_{n}^{\left(q\right)}\left(\tilde{t}\right) & =a^{\left(q\right)}e^{j2\pi R_{0}^{\left(q\right)}\left(-\frac{2tB}{cT}-\frac{2f_{c}+B}{c}\right)}\times e^{j2\pi v^{\left(q\right)}\left(-\frac{2f_{c}+B}{c}mT-\frac{2mtB}{c}\right)}\\ \; & \times e^{j2\pi \tau_{An}^{\left(q\right)}\left(\frac{-Bt}{T}-f_{c}-\frac{B}{2}\right)}\times e^{j2\pi \tau_{An}^{\left(q\right)}R_{0}^{\left(q\right)}\frac{2B}{cT}}\\ \; & \times e^{j2\pi \tau_{An}^{\left(q\right)}v^{\left(q\right)}\frac{2Bm}{c}}\times e^{j2\pi {\left(\tau_{An}^{\left(q\right)}\right)}^{2}\frac{B}{2T}}\times e^{j2\pi \left(Bt+f_{c}T\right)}. \end{array}$$

We can notice that mixed terms appear in the expression for the dechirped signal, which clearly shows that there is a certain coupling between range, Doppler and azimuth domain. The terms standing next to the $R_{0}^{\left(q\right)}$ in the previous two equations correspond to the range of the *q*-th target. The terms standing next to the $v^{\left(q\right)}$ correspond to Doppler effect because Doppler frequency $f_{d}^{\left(q\right)}$ can be expressed using radial velocity as $f_{d}^{\left(q\right)}=2v^{\left(q\right)}f_{c}/c$, and normalized Doppler frequency is $\mu_{q}=\left(2\pi T\right)2v^{\left(q\right)}f_{c}/c$. In addition, the terms standing next to the $\tau_{An}^{\left(q\right)}$ correspond to the azimuth of the *q*-th target, because the delay at the *n*-th receive antenna w.r.t. the referent point of the receiving array can be expressed as $\tau_{An}^{\left(q\right)}=\left(n-1\right)d\sin\theta^{\left(q\right)}/c$, where *d* represents the distance between antenna elements of the receiving array and $\theta^{\left(q\right)}$ is the azimuth of the target.

In the Rx, the received signal is given by

(13) $$y_{n}\left(\tilde{t}\right)=\eta_{n}\left(\tilde{t}\right)+\sum_{q}y_{n}^{\left(q\right)}\left(\tilde{t}\right),$$

where ionospheric interference, sea clutter and additive noise are modeled by $\eta_{n}\left(\tilde{t}\right)$, and the sum is over all the targets.

Finally, the dechirped signal is sampled at a rate $f_{s}$ in the A/D converter.

## 3. Detailed Algorithm Description

**Figure 3:**
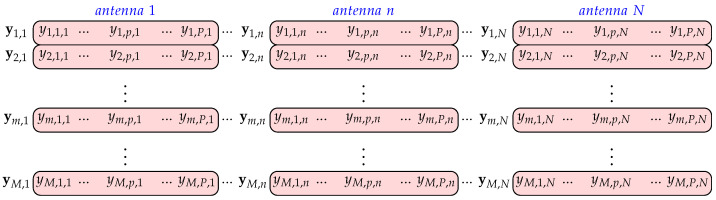
Three-dimensional matrix **Y** with complex signal samples (*M* frames from *N* antennas) from the output of the dechirper.

A three-dimensional matrix with acquired complex time samples of IQ branch signals at the receiving channels is denoted by $Y\in C^{M\times P\times N}$, whose elements are

(14) $$y_{m,p,n}=y_{n}\left(\left(m-1\right)T+\left(p-1\right)/f_{s}\right),$$

for 1≤m≤M, 1≤p≤P, 1≤n≤N. In practical situations, *P* and *N* are predefined values and *M* is the value to be chosen and it should correspond to the integration period in which signal coherence is preserved. The developed algorithms were tested for the length of the segment of M=256, where the successive segments overlap in 128 frames. This ensures that the results are ejected every 128 frames. RAW data were recorded with 2048 frames per file. In the analysis, the acquired frames are divided into 15 overlapping segments, each with 256 overlapping frames. A vector with *P* complex signal samples at the *n*-th antenna in the *m*-th frame is defined as $y_{m,n}=\left[y_{m,1,n},\ldots ,y_{m,p,n},\ldots ,y_{m,P,n}\right]\in C^{1\times P}$, where $y_{m,p,n}$ denotes the *p*-th complex signal sample at the *n*-th antenna in the *m*-th frame (segment). Figure 3 shows Y matrix.

**Figure 4:**
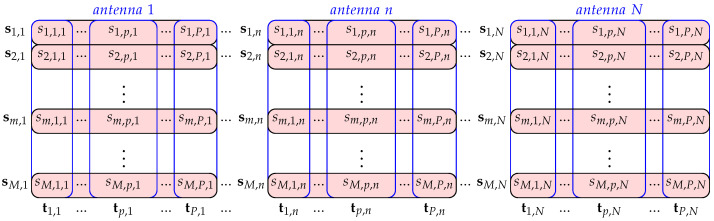
Matrix **S** formulation.

The first step in classical processing in many radars, such is WERA radar, is the implementation of FFT algorithm of vectors $y_{m,n}$ for all frames m=1,2,…,M and all antennas n=1,2,…,N, and thus obtaining a three-dimensional matrix $S\in C^{M\times P\times N}$ with complex samples of the spectrum whose rows $s_{m,n}\in C^{1\times P}$ represent vectors with the spectrum samples in the *m*-th frame and at the *n*-th antenna, which are obtained as:

(15) $$s_{m,n}=\left(w_{P}⊙y_{m,n}\right)F_{P},$$

where $w_{P}=\left[w_{1},w_{2},\ldots ,w_{P}\right]\in R^{1\times P}$ denotes the vector with the samples of the applied Blackman–Harris window function, the operator (⊙) denotes Hadamard’s (Shur’s) product and $F_{P}\in C^{P\times P}$ denotes the Fourier matrix of P×P dimensions. The second step in the classic primary processing of many radars is the implementation of the FFT algorithm per matrix S columns $t_{p,n}={\left[s_{1,p,n},\ldots ,s_{m,p,n},\ldots ,s_{M,p,n}\right]}^{⊤}\in C^{M\times 1}$ for all antennas n=1,2,…,N and all samples p=1,2,…,P per frame as follows:

(16) $$h_{p,n}^{⊤}=\left(w_{M}⊙t_{p,n}^{⊤}\right)F_{M}\in C^{1\times M},$$

where $w_{M}=\left[w_{1}^{\prime },w_{2}^{\prime },\ldots ,w_{M}^{\prime }\right]\in R^{\left(1\times M\right)}$ denotes samples of the applied Blackman–Harris window function, and ${\left(\cdot \right)}^{⊤}$ is transpose operation. The vectors $h_{p,n}$ are calculated for p=P−R+1,P−R+2,…,P, where *R* corresponds to the maximum projected radar range. The matrix $F_{M}\in C^{M\times M}$ denotes the Fourier matrix of M×M dimensions. Figure 4 shows S matrix with selected vectors used in the previous analysis.

A matrix $H_{n}\in C^{M\times R}$ is then formed as follows:

(17) $$H_{n}=\left[h_{P-R+1,n},h_{P-R+2,n},\ldots ,h_{P,n}\right]\in C^{M\times R}.$$

The matrix $H_{n}$ represents the Range–Doppler matrix at the *n*-th antenna. This matrix is obtained using the FFT algorithm and we’ll call it the RD-FFT map. A further procedure of primary signal processing in some existing OTHR radars, such is WERA, is CFAR detection of targets in the RD-FFT map, followed by the estimation of signal arrival direction for detected targets using classical single snapshot beamforming.

### 3.1. High-Resolution Range–Doppler (RD-HR) Map Estimation (Uniform Sampling Method in Slow Time Domain)

**Figure 5:**
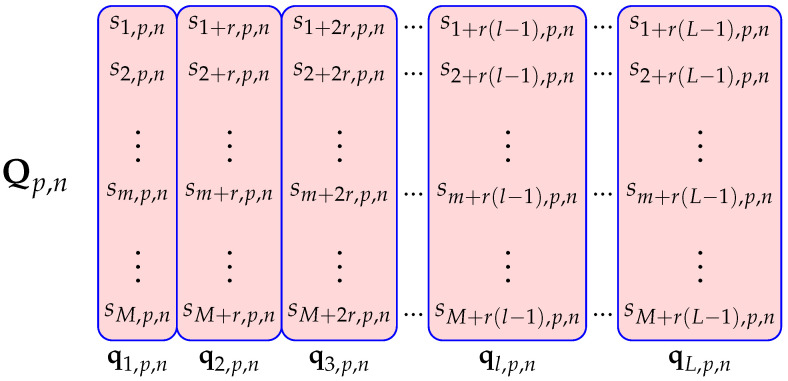
The creation of $Q_{p,n}$ matrix from $S_{E}$ matrix.

The high-resolution algorithm for creating the RD-HR map starts from the formation of the matrix $S\in C^{M\times P\times N}$ using Fourier transform. By adding zeros to the vectors with signal samples, better computational (but not actual) resolution can be achieved when applying FFT. Thus, the proposed algorithm for creating the RD-HR map is computationally high-resolution in the range domain and essentially computationally high-resolution in the Doppler domain. From the original matrix S we form an extended matrix $S_{E}\in C^{(M+r(L-1))\times P\times N}$ by adding r(L−1) additional frames. Then new matrices $Q_{p,n}\in C^{M\times L}$ are formed for 1≤p≤P and 1≤n≤N, whose columns are vectors $q_{l,p,n}$, 1≤l≤L. A total of M+r(L−1) frames are used to form this matrix. The index *r* denotes the number of frames that do not overlap in adjacent vectors $q_{l,p,n}$. The developed algorithm was tested for r=1. The reason for this is a better estimate of the covariance matrix later. Figure 5 shows the creation of $Q_{p,n}$ matrix from $S_{E}$ matrix.

Then the covariance matrices $C_{p,n}\in C^{M\times M}$ are formed for n=1,2,…,N and p=P−R+1,P−R+2,…,P as follows:

(18) $$C_{p,n}=\frac{1}{L}Q_{p,n}Q_{p,n}^{H}\in C^{M\times M}.$$

The matrix $C_{p,n}$ is a complex square Hermitian (conjugate symmetric) positive definite matrix, which means that the eigenvalues of that matrix are positive quantities. The formation of the covariance matrix $C_{p,n}$ is a key step in the formulation of the high-resolution algorithm for the creation of the RD-HR map. Based on the covariance matrix, it is possible to formulate several criterion functions of high-resolution algorithms for creating an RD-HR map. In this case, we use MUSIC-based algorithm, as follows:

(19) $$P_{\mathrm{MUS}}^{\mathrm{RD}}\left(\mu ,p,n\right)=\frac{1}{∥a_{\mu }{\left(\mu \right)}^{H}E_{p,n}∥}.$$

The matrix $E_{p,n}\in C^{M\times (M-K)}$ is a matrix of noise subspace of the covariance matrix $C_{p,n}$ whose columns are eigenvectors of the covariance matrix $C_{p,n}$ which correspond to M−K of the smallest eigenvalues of the covariance matrix $C_{p,n}$, where *K* represents a parameter of the MUSIC-based algorithm. As is well known, the MUSIC algorithm requires the knowledge of the dimensionality of the signal subspace (parameter *K*). There are several algorithms in the literature that can be used to estimate *K*, such as the Minimum Description Length algorithm (MDL) [41] or the Akaike Information Criteria (AIC) [46]. Those algorithms work well in controlable signal scenario, such as a simulation. However, from our previous experience, we know that in real coherent systems (such as direction finder we developped) these algorithms tend to overestimate the value of *K*. It is known from the theory of subspace algorithms, that when the eigenvalues are sorted in descending order, there is an inflection point in the curve whose position indicates the dimensionality of the signal and the complementary noise subspace. The eigenvalues of the covariance matrix, whose values are approximately equal to the variance of the noise on receiving channels, determine the dimensionality of the noise subspace. So, we analyzed empirically the position of that inflection point and observed that the *K* values 5 and 10 produce satisfactory results compared with AIS. However, an adaptive algorithm should be applied for the estimation of *K*. Also, the term coherence we use in two contexts. The first is related to a multipath environment where multiple delayed replicas of the same signal are superposed on the antenna array. This is known to be a problem for most algorithms in array processing. The second is related to the period of integration. The choice of the integration period implicitly assumes that in this period of time the Doppler shift of the signal due to the movement of the target is constant.

The vector $a_{\mu }\left(\mu \right)\in C^{M\times 1}$ is an equivalent steering vector formulated in the normalized Doppler domain as:

(20) $$a_{\mu }\left(\mu \right)={\left[1,e^{-j\mu },\ldots ,e^{-j\mu (M-1)}\right]}^{⊤},$$

where the parameter μ denotes the normalized Doppler frequency in radians per frame. The criterion functions are calculated for a set of discrete values of normalized Doppler frequencies, for the range of Doppler frequencies of interest and with a resolution that is many times better than FFT resolution per Doppler, thus obtaining a zoomed high-resolution RD-HR map. With RD-HR maps obtained by MUSIC method, the high-resolution properties and better detectability and contrast of targets in the RD-HR map in relation to the RD-FFT map are clearly observed. Range and Doppler frequencies are estimated by detecting peaks in the RD-HR map. The arguments of the maxima of these peaks are $\left(\mu_{q},p_{q}\right)$, where $1\leq q\leq N_{d}$, $N_{d}$ is the total number of detected peaks and $\mu_{q}$ and $p_{q}$ correspond to Doppler and range domain, respectively. For each of the detected $N_{d}$ peaks, the direction (azimuth) of the target is then estimated. For high-resolution azimuth estimation, the same type of criterion function is used as for RD-HR map estimation, with the difference that steering vectors and covariance matrices are formed in the spatial domain by *n* and for the detected indexes $\mu_{q}$ and $p_{q}$ in Doppler and range domain, respectively.

### 3.2. High-Resolution Range–Doppler (RD-HR) Map Estimation (Non-Uniform Sampling Method in Slow Time Domain)

It was found that the covariance matrix $C_{p,n}$. For all *n* and *p* has a conditional number of order $10^{19}$ which means that it is close to the singular matrix which can in some cases result in problems of numerical instability during inversion and eigenvalue decomposition of the matrix. Known procedures for reducing the conditional number are analyzed (such as adding a small scalar value on the diagonal of the matrix $C_{p,n}$. The motivation for formulating a high-resolution algorithm for estimating the RD-HR map with non-uniform sampling is related to reducing the numerical complexity of the matrix algorithm and to reducing the conditional number of the matrix of that matrix. In uniform sampling method, the dimensionality of the covariance matrix is M×M. The idea of non-uniform sampling is that dimensionality of the covariance matrix be smaller (J×J) where J<M. The problem of non-uniform sampling method in this case is analogous to the problem formulated in the field of antenna arrays - how to replace a linear uniform antenna array with a non-uniform antenna array with the same aperture and a smaller number of antennas without significant degradation of the antenna array factor? This problem in antenna array theory is known as the problem of minimally redundant linear antenna arrays. High-resolution algorithm for the RD-HR matrix creation therefore has its theoretical foundation in the theory of antenna arrays or array processing.

The idea is to select a subset of *J* rows of the matrix $Q_{p,n}$ by choosing an appropriate mapping $\ell :\left\{1,2,\ldots ,J\right\}\to \left\{1,2,\ldots ,M\right\}$, J<M. We then form $Q_{p,n}^{\left(\ell \right)}$ as

(21) $${\left[Q_{p,n}^{\left(\ell \right)}\right]}_{j,l}={\left[Q_{p,n}\right]}_{\ell (j),l},$$

where ${\left[A\right]}_{j,l}$ denotes the $\left(j,l\right)$-th element of A. Thus we form a new matrix $Q_{p,n}^{\left(\ell \right)}\in C^{\left(J\times L\right)}$ on the principle of non-uniform selection of the elements of the column vector of the matrix $Q_{p,n}$. The same mapping *ℓ* is used to form the steering vector $a_{\mu }^{\left(\ell \right)}\left(\mu \right)$ by non-uniform selection of the elements of the vector $a_{\mu }\left(\mu \right)$. Thus, we get a much smaller covariance matrix. Covariance matrices $C_{p,n}^{\left(\ell \right)}\in C^{\left(J\times J\right)}$ are formed for n=1,2,…,N and p=P−R+1,P−R+2,…,P as follows:

(22) $$C_{p,n}^{\left(\ell \right)}=\frac{1}{L}Q_{p,n}^{\left(\ell \right)}Q_{p,n}^{H\;(\ell )}.$$

The criterion function of the high-resolution MUSIC-type algorithm for creating RD-HR map with non-uniform sampling has the same form as the criterion function for the variant with uniform sampling (with the noise subspace matrix and steering vector in the Doppler domain being formed as described above) and defined as:

(23) $$P_{\mathrm{MUS}}^{\mathrm{RD}(\ell )}\left(\mu ,p,n\right)=\frac{1}{∥a_{\mu }^{\left(\ell \right)}{\left(\mu \right)}^{H}E_{p,n}^{\left(\ell \right)}∥}.$$

$E_{p,n}^{\left(\ell \right)}$ is obtained from $C_{p,n}^{\left(\ell \right)}$ in the same way $E_{p,n}$ is obtained from $C_{p,n}$ in the uniform sampling method by selecting the elements according to the same mapping *ℓ*.

The procedure for detection and high-resolution estimation of direction (azimuth) is further identical as in the case of the variant of the algorithm with uniform sampling.

### 3.3. Detection of Targets on the RD-HR Map

The complete procedure, presented in the previous part of this chapter, is used to evaluate the high-resolution RD-HR (Range–Doppler High Resolution) map. The Range–Doppler map in this document is defined as a numerical two-dimensional image consisting of a finite number of points, which provide accurate information on activity at sea. It is formed at all antennas and for all data segments. The high-resolution properties of the algorithm contribute to better ship detectability, as well as the ability to detect some ships, which are not visible at all using the currently used primary signal processing algorithms. So, the precondition for detection is the formation of RD-HR map, which is the most computationally demanding part of this algorithm.

Comparing the RD-FFT and the RD-HR map clearly shows the advantages of the RD-HR map in terms of resolution properties, map contrast and the ability to detect targets, which fully justifies the use of the algorithm. It was also noticed that successive detections of ships of interest are chained, passing through all segments, and that there is an overlap of contours of criterion functions of successive detections, which is the basis for formulating criteria for detection consistency and a step towards an improved version of tracking.

In this regard, we will describe the procedure for detecting targets (ships) from the RD-HR map using a newly developed algorithm for Image Processing [27]. The algorithm is innovative and adapted to the specifics of this type of Range–Doppler maps (characteristics of peaks in their criterion functions). Therefore, a joint estimation of the ship’s distance and its Doppler frequency is performed, based on finding peaks at the RD-HR map according to a precisely defined criterion. At the beginning of the detection process, we have to determine arithmetic mean of RD-HR maps at all antennas. Thus, we’ll have only one RD-HR map (criterion function) that is used to find detections:

(24) $${\bar{P}}_{\mathrm{MUS}}^{\mathrm{RD}}\left(\mu ,p\right)=\frac{1}{N}\sum_{n=1}^{N}P\left(\mu ,p,n\right),$$

where P(μ,p,n) can be uniform or non-uniform criterion function obtained by choosing appropriate mapping *ℓ*. From the criterion function ${\bar{P}}_{\mathrm{MUS}}^{\mathrm{RD}}\left(\mu ,p\right)$ we form the matrix $P_{\mathrm{MUS}}^{\mathrm{RD}}$ which consists of elements of interest ($1\leq \mu \leq M_{P},1\leq p\leq R$). $M_{P}$ and *R* are lengths of RD-HR map by Doppler and range dimension, respectively. The grid resolution is 4 times better for range and 2 times better for Doppler than in the case of FFT map.

The next step in the detection procedure is to analyze the RD-HR map (data matrix) and numerically present it using 16 bits. This actually means that the RD-HR map (2D image) is represented by $2^{16}$ different values, and that the minimum value of the RD-HR map criterion function is zero and the maximum value is 65,535. The reason for this conversion is the higher speed of ship detection, during the practical implementation of this algorithm, without losing the image quality at all in terms of poorer detection.

The next procedure is to remove unwanted noise from the image (RD-HR map) which is presented as a relatively high value in the vicinity of which are relatively low values. This type of noise would manifest itself in the picture as a single point (of course, unwanted in this case). In practice, this would mean that this point would represent a peak in the criterion function, and later also the detection, which we already know in advance is not a real detection, but a false alarm. In the literature, this type of forest is known as “Salt and Pepper” and its elimination is required. The reason why these values not be considered as detection lies in the analysis of peaks at the RD-HR map. It has been experimentally observed that the peaks are not point values, but that they have some shape everywhere, whose width is smaller or larger, and their values of the criterion function cannot drastically fall at all neighboring points around one peak.

The elimination of unwanted values is done by Median Filtering algorithm used in Image Processing. This filtering method is executed for each element of the input matrix (each pixel of the input map), analyzes its 8 adjacent values, and calculates the median of these nine values. The procedure is as follows:

1. A window (matrix) of size 3×3 points is placed around the observed image element (at the beginning it starts from the upper leftmost point in the image, ie the first row and the first column);
2. Then all the elements in the window are placed in an array;
3. The array is sorted in ascending order;
4. Select the mean element (5-th element in the array);
5. The corresponding image element, which has been filtered, is written to the appropriate element of the output matrix (the pixel of the output map);
6. The procedure steps 1–5 are repeated for each element of the output matrix.

**Figure 6:**
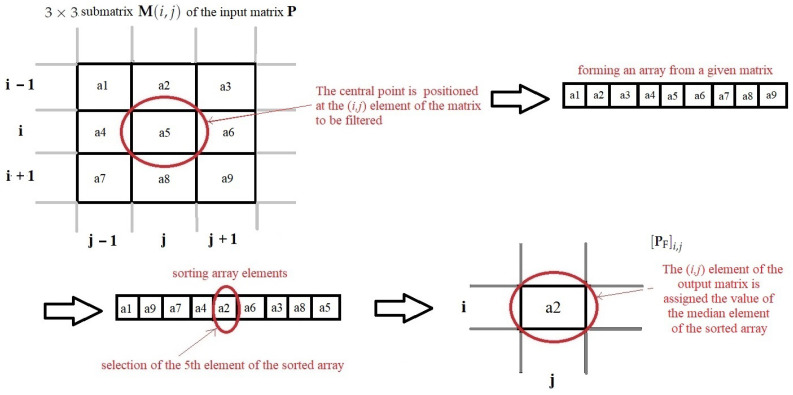
Median Filtering algorithm.

It is necessary to emphasize that the filtering window can be of different lengths, but in this particular case it is necessary to remove only unwanted single-point peaks from the image, because we know for sure that they do not represent detection. Setting up a larger window would not be practical, as there is a possibility that the criterion function of the Range–Doppler map would be very narrow, and in that case it would be unreasonably rejected, which would lead to poorer detection results. Also, it should be emphasized that the algorithm is applied to points located on the edges of the image, and all points of the window function, which can not include the nearest neighbors of the filtered point, are simply supplemented by zeros and the median is sought, as which has been explained before. There are several cases when zeros are filled in the window function, and they are: first row, first column, last row and last column of the Range–Doppler map. Because of this, there will certainly be zeros at all ends of the filtered image. In Figure 6, the procedure of the Median Filtering algorithm was shown.

In other words, for a given map P, we select a 3×3 submatrix M(i,j) centered at (i,j) as

(25) $$M\left(i,j\right)={\left[P\right]}_{i-1:i+1,j-1:j+1}\;\left(\forall i,j\right),$$

where each edge of the map is padded with zeros, or more formally, ${\left[P\right]}_{i,j}=0$, for all i,j where $i\in \left\{0,M_{P}+1\right\}$ or $j\in \left\{0,R+1\right\}$. P can be $P_{\mathrm{MUS}}^{\mathrm{RD}}$ in this case. $M_{P}$ and *R* are lengths of RD-HR map by range and Doppler dimension, respectively.

Then the submatrix is rearranged into a vector, $m\left(i,j\right)=\mathrm{vec}\left\{M(i,j)\right\}\in R^{9\times 1}$, the vector is sorted which produces the vector $m_{s}\left(i,j\right)$, and the resulting pixel is ${\left[P_{F}\right]}_{i,j}={\left[m_{s}\left(i,j\right)\right]}_{5}$.

These steps are executed for each pixel (*i*,*j*) of the RD-HR map in order to eliminate spurious single-pixel peaks.

**Figure 7:**
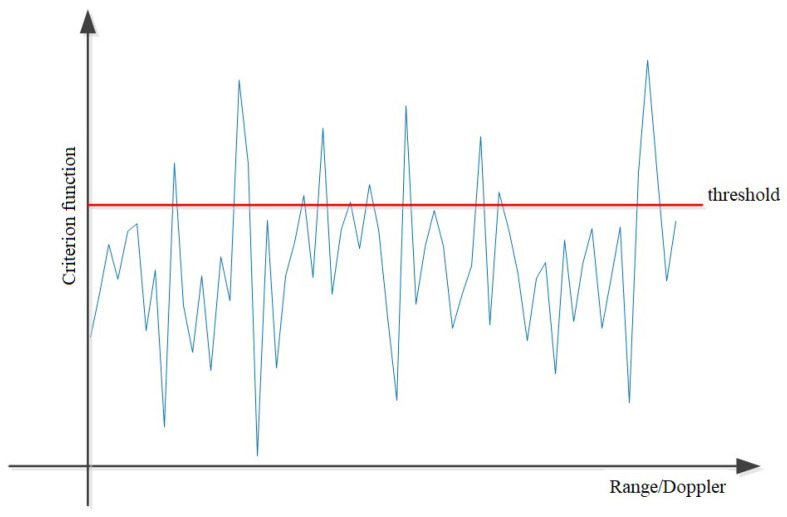
Detection procedure using threshold.

Next, the value of the threshold above which detections are taken is defined. So, the threshold value is a parameter that determines whether some peaks in the criterion function will be considered as detections or noise (such detections are rejected). The selection of the threshold value is an important procedure during detection and it is necessary to pay special attention to it. Its value can vary depending on whether the detection of close or distant ships is desired, and it can also be an adaptive threshold value depending on the distance. Figure 7 shows the ship detection procedure, whose values of the criterion function are above the threshold.

From a numerical point of view, we wonder what that threshold value is. If a low threshold is chosen, the number of detections will be higher, and all ships will be detected, both those that are close, but also those that are very far from the radar. In that case, the number of detections will be large, but this increases the complexity of the calculation later, when we are detecting the azimuth. It has been noticed that there are a large number of false alarms among these detections, so the question is how the threshold can be set. To choose the appropriate value for the threshold, the amplitude of the targets have to be taken into account. The greater the amplitude (on average), the higher the threshold value should be, otherwise the false alarm rate would be increased. If, on the other hand, the amplitude is low and we do not decrease the threshold adequately, the probability of misdetection would increase. The amplitude of the signal generally decreases with the range. Additionally, it increases with the increase of the target RCS. All in all, an adaptive threshold strategy should be implemented, so that the threshold value is nonuniform along the range dimension. Also, the threshold value should be increased in the immediate vicinity of a target with a large RCS, to keep the false alarm rate down. Based on experimental tests of data obtained from radar in operational work, it was concluded that the threshold value should be in the range of $0.1\times 2^{16}$ and $0.2\times 2^{16}$, while the number of successful detections should be maximum. In the implementation process of the algorithm, the values 0.1 and 0.2 are used, thinking about the previously defined values $0.1\times 2^{16}$ and $0.2\times 2^{16}$. In this study, the threshold values were chosen according to a large set of real radar data (large statistical sample) and applied within the algorithm to obtain the target detections. In this paper we present a small subset of these results. Of course, it should be noted that not all points that exceed the threshold value will be detections, but they will become candidates to be detections, which will be discussed later.

**Figure 8:**
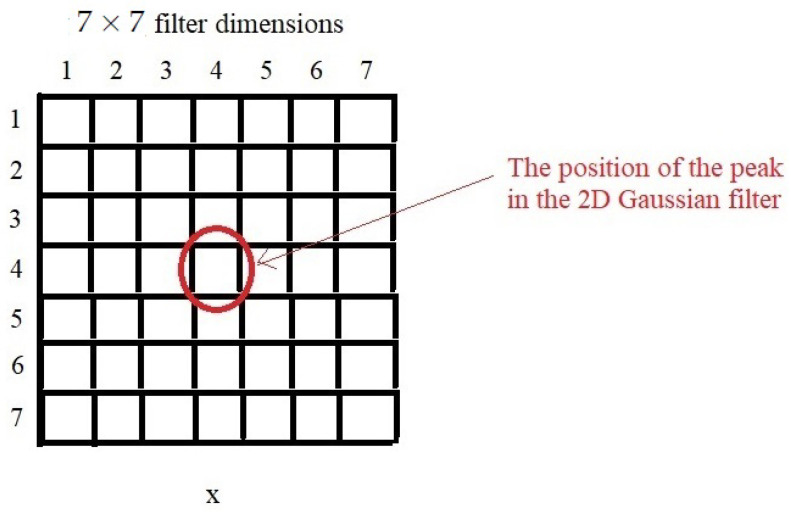
Gaussian 2D filter of dimensions 7×7.

The next step is the 2D convolution procedure. Here, the RD-HR map (2D image) is additionally filtered, but there must be another 2D filter matrix of smaller dimensions (the so-called kernel matrix).The main goal is to obtain a smooth image Range–Doppler map. The filter matrix in this case is a Gaussian 2D filter whose dimensions are 7×7. The dimensions of this filter can be different, but it would be best to choose them based on the characteristics of the RD-HR map peaks. The center of the filter matrix must be positioned on the pixel to be filtered. This operation in which we summarize the products of the elements of two 2D functions, where it is allowed for one of the two functions to move over the elements of the other function is actually a convolution. The 2D convolution operation is quite computationally demanding, so it is not very fast to execute unless small kernel filters are used. Their dimension should be odd, so that they have a center, for example 3×3, 5×5 and 7×7. In Figure 8 it is shown a Gaussian 2D filter measuring 7×7, as well as its criterion function.

**Figure 9:**
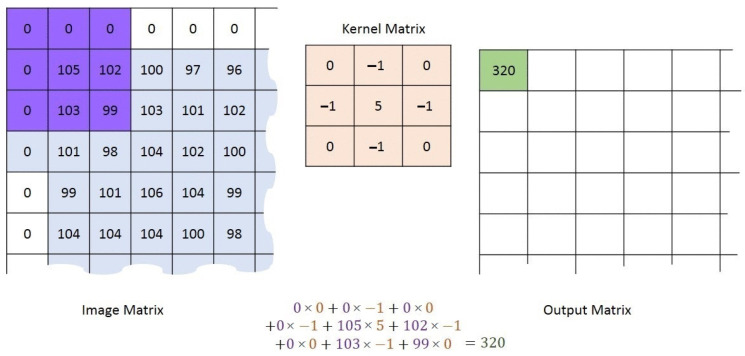
2D convolution process with a 3×3 kernel matrix for one pixel at the RD-HR map.

Figure 9 illustrates the 2D convolution procedure with a 3×3 kernel matrix for one pixel at the RD-HR map. As explained earlier, the same is true here if the dot is found on the edges of the RD-HR map, then the corresponding values of the kernel matrix are filled with zeros.

**Figure 10:**
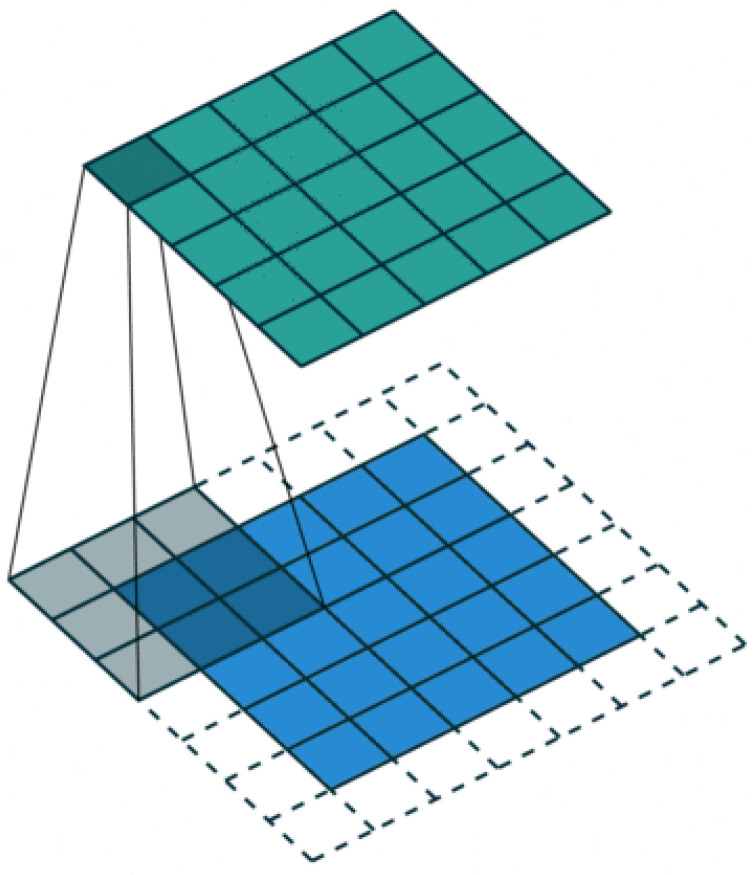
2D convolution process.

Figure 10 illustrates the 2D convolution procedure with a kernel matrix, which clearly shows how to process one pixel at a time from the image.

The last step is to determine the actual detections, based on the matrix obtained after the procedure of all the above filtering and with a defined value of the detection threshold. It should be noticed that not all values obtained in this way. First, non-zero elements are determined (which are significantly smaller than in the original image), and they represent candidates for detection. Then, around each point, a criterion function is observed with 2 points on all sides.

In other words, we want to apply a 2D linear FIR (Finite Impulse Response) filter to the map $P_{F}$ to obtain $P_{\mathrm{FF}}$. Its impulse response (or kernel) can be thought of as a 7×7 matrix and is given by

(26) $$\kappa \left(i,j\right)=\frac{1}{\sigma^{2}}\exp\left(-\frac{i^{2}+j^{2}}{2\sigma^{2}}\right);\left(\forall i,j\in \left\{-3,-2,\ldots ,3\right\}\right).$$

The result is the convolution

(27) $${\left[P_{\mathrm{FF}}\right]}_{i,j}=\sum_{\zeta =-3}^{3}\sum_{\xi =-3}^{3}\kappa \left(\zeta ,\xi \right){\left[P_{F}\right]}_{i-\zeta ,j-\xi }$$

where the edges of the map $P_{F}$ are appropriately padded with zeros, i.e., ${\left[P_{F}\right]}_{i,j}=0$, for all i,j where $i\in \left\{-2,-1,0,M_{P}+1,M_{P}+2,M_{P}+3\right\}$ or $j\in \left\{-2,-1,0,R+1,R+2,R+3\right\}$. Kernels of different sizes, such as 9×9, 5×5, or 3×3, can be used instead, but filtering with large kernels can be computationally demanding.

**Figure 11:**
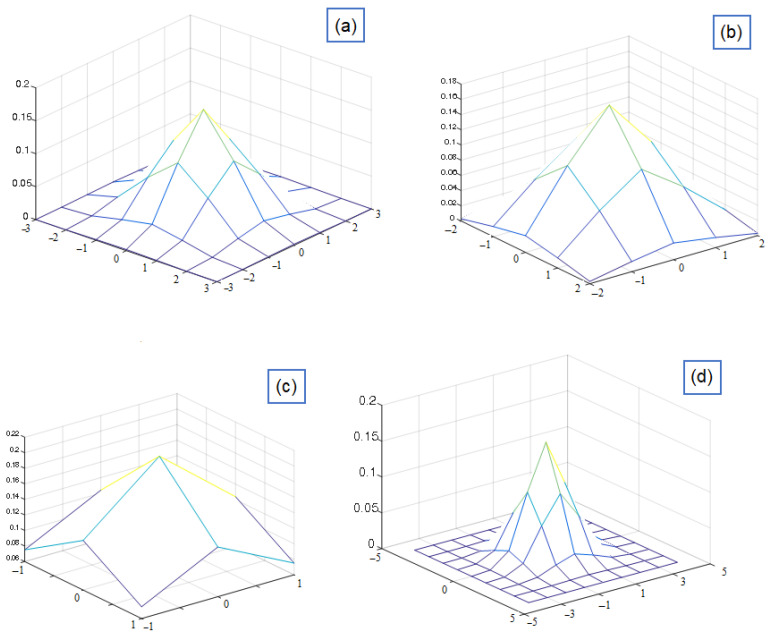
The layout of different kernel functions: (**a**) 7×7 (**b**) 5×5 (**c**) 3×3 (**d**) 9×9.

Figure 11 shows the layout of different kernel functions.

There are 2 ways to search for the appropriate detection. The first way is the method of local maximum of the criterion function, more precisely, if the point, which is located in the center of the part of the criterion function of dimensions 5×5, has a maximum value in that window, then it represents detection. The second way involves the analysis of the same part of the criterion function, but also the sigma by distance and Doppler frequency. Sigma, as it was defined in WERA radar, means 2nd moments of the distance and Doppler estimation, while the distance and Doppler estimates are centroids (centers of mass) of this part of the criterion function. The result of the execution of the algorithm are detections, whose x and y coordinates represent estimates of the distance and Doppler frequency.

**Figure 12:**
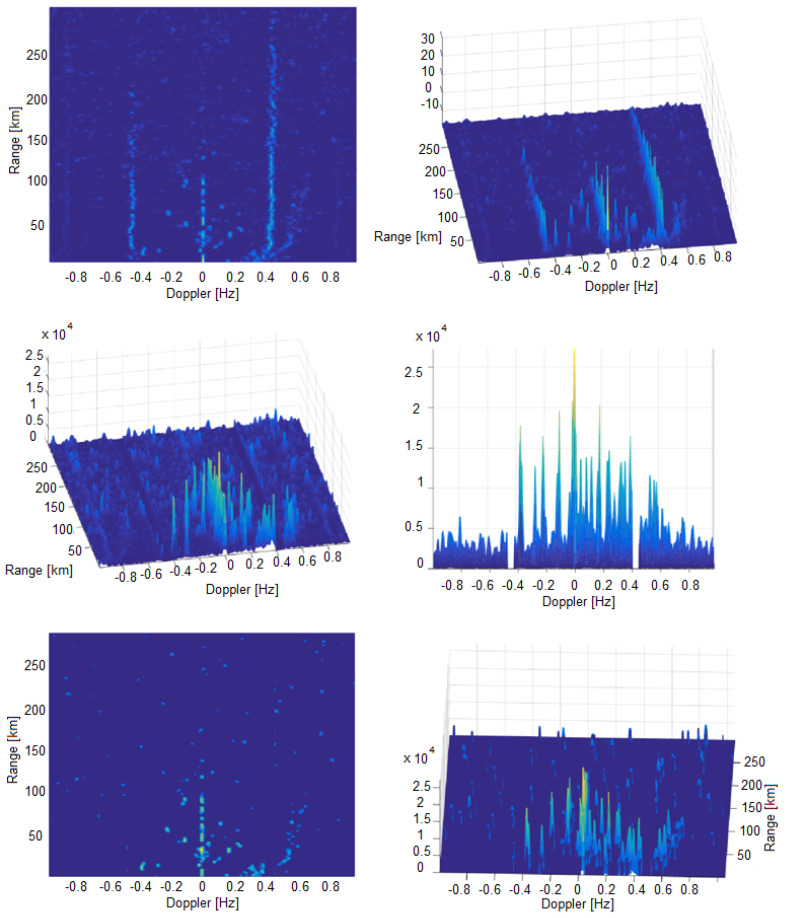
RD-HR map at the beginning of the process (**top**), RD-HR map after median filtering and Bragg’s lines elimination (**middle**) and HD HR map after convolution process (**bottom**).

Figure 12 shows all steps in detection process.

**Figure 13:**
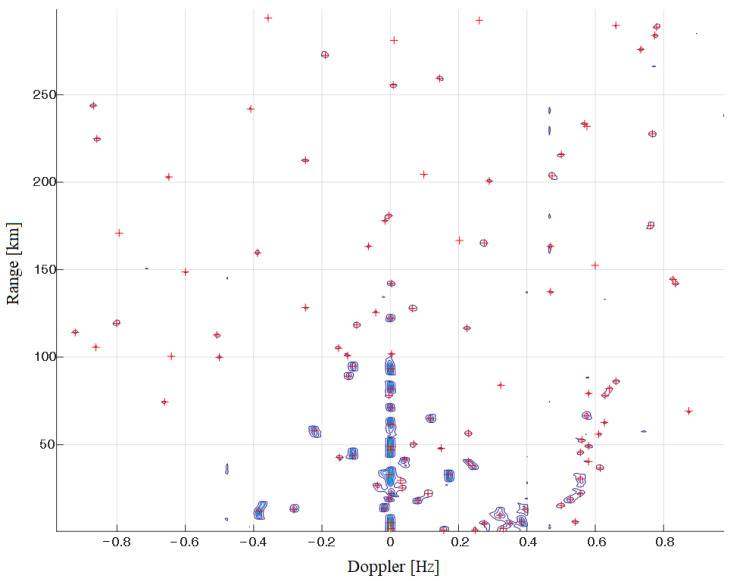
Detections on RD-HR map (detections are denoted by “+” markers, and the blue contours represent the contours of the criterion function of the MUSIC-based algorithm).

Finally, we obtain detections on Range–Doppler map and we can continue with the last step in the detecion process which is azimuth detection. Figure 13 shows detections on RD-HR map.

### 3.4. Azimuth Detection of Targets Detected in RD-HR Map

The idea is to first evaluate the high-resolution RD-HR map, to detect targets on it using a newly developed algorithm for Image Processing, and to estimate the azimuth, using another algorithm. Azimuth detection performs only for distances and Doppler frequencies of such detected targets. In this way, the numerical complexity is significantly reduced and the execution process of the algorithm is accelerated. For the calculation of azimuth, a similar procedure will be used as before. For detected distances, a criterion function will be required, but only for detected Doppler frequencies. The MUSIC method will give an accurate azimuth estimation.

**Figure 14:**
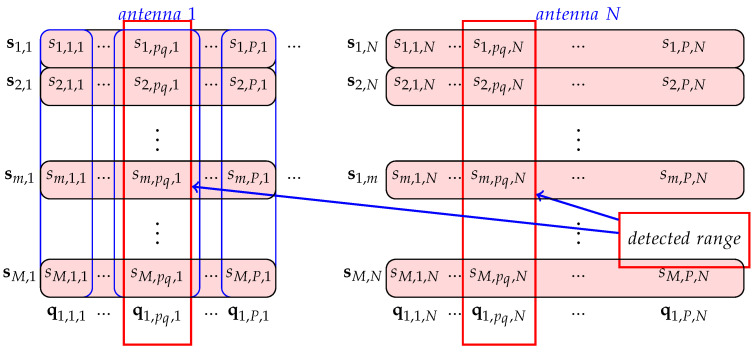
Finding of the appropriate column vector $q_{l,p_{q},n}^{\left(q\right)}$ from FFT matrix for detected range $p_{q}$ and l=1.

We will assume that the number of detections found on the Range–Doppler map is equal to $N_{d}$. Therefore it is necessary to determine the unknown parameters $\mu_{q},p_{q}$ and $\theta_{q}$ or the unknown vessel Doppler frequencies (radial velocities), ranges and directions of arrival (azimuths) for each of $q=1,2,\ldots ,N_{d}$ targets, respectively. Unlike the RD-FFT map, when RD-HR map is formed, the phase data is lost and therefore we have to return to the matrix $S_{E}$. The first step in azimuth detection is to select column vectors $q_{l,p_{q},n}$ for 1≤l≤L, 1≤n≤N and for $p_{q}$-th FFT sample. These columns correspond to the detected range from the RD-HR map. Thus, for each of *q* detections we find the appropriate column vectors $q_{l,p_{q},n}$, as shown in Figure 14.

To compensate for the Doppler effect in matrix $Q_{p_{q},n}$ for all antennas n=1,2,…,N, we use the steering vector $a_{\mu }\left(\mu_{q}\right)$ and we get:

(28) $$r_{n}^{\left(q\right)}=\left(w_{M}⊙a_{\mu }{\left(\mu_{q}\right)}^{H}\right)Q_{p_{q},n}\in C^{1\times L}.$$

Then we form appropriate matrix

(29) $$R^{\left(q\right)}={\left[r_{1}^{(q)⊤},r_{2}^{(q)⊤},\ldots ,r_{N}^{(q)⊤}\right]}^{⊤}\in C^{N\times L}.$$

We get the covariance matrix by averaging over the *L* shifts as follows

(30) $$C_{A}^{\left(q\right)}=\frac{1}{L}R^{\left(q\right)}R^{{\left(q\right)}^{H}}\in C^{N\times N}.$$

The same snapshots/frames ($Q_{p,n}$ from Figure 5) that are used for the estimation of the covariance matrix (18) of the RD-HR map are also used for the estimation of the covariance matrix in (30), after pre-processing according to (28). When selecting the parameter *L*, a compromise must be made here because on one hand, to allow a more statistically stable estimate of the covariance matrix, a large enough number of snapshots should be taken, but on the other hand it increases the numerical complexity, so the signal processing cannot be performed in real-time. Another negative effect of increasing *L* too much is that it can become longer than the coherence interval and, so, will blur the RD-HR map. In the practical implementation, L=64 is the value that satisfies all the above requirements.

The steering vector in this case is

(31) $$a_{\theta }\left(\theta \right)={\left[1,e^{-j\nu },\ldots ,e^{-j\nu (N-1)}\right]}^{⊤}.$$

for a ULA, where $\nu =2\pi f_{c}d\sin\theta /c$, θ is the azimuth, and *d* is the distance between adjacent antennas. The criterion function for the azimuth is

(32) $$P_{\mathrm{MUS}}^{A}\left(\theta ,q\right)=\frac{1}{∥a_{\theta }^{H}\left(\theta \right)E^{\left(q\right)}∥},$$

where $E^{\left(q\right)}$ is the noise subspace matrix calculated for the *q*-th detected target. The matrix $E^{\left(q\right)}\in C^{N\times (N-K_{A})}$ is a matrix of noise subspace of the covariance matrix $C_{A}^{\left(q\right)}$ whose columns are eigenvectors of the covariance matrix $C_{A}^{\left(q\right)}$ which correspond to $N-K_{A}$ of the smallest eigenvalues of the covariance matrix. $K_{A}$ represents a parameter of MUSIC-based algorithm.

The final estimate of the azimuth is determined by:

(33) $${\hat{\theta }}^{\left(q\right)}=\underset{\theta }{arg max}\left|P_{\mathrm{MUS}}^{A}\left(\theta ,q\right)\right|.$$

## 4. Experimental Results

The results presented in this section are based on the measured radar data, and their verification was made using AIS data. A set of real signals (RAW data) acquired on April 19, 2020 from the OTHR radar located on Ibeju Lekki, Nigeria, in a time interval of 5 h was used for testing. The section is divided in 2 parts.

The first part of this section shows Range–Doppler maps obtained by the proposed HR algorithm, and one example of azimuth estimation based on detections from RD-HR maps. In this part, the basic system parameters are explained too. The second part analyzes the performance of the proposed algorithm. Because we have real data and also AIS data, we can made one experiment to see the numerical results of detecting vessels.

P = 1536 and N = 16 are predefined values and M is the value to be selected and it should correspond to the integration period in which the coherence of the signal is preserved. The developed algorithm was tested for the length of the segment M = 256, where the successive segments overlap with 128 frames, which ensures the results are ejected every 128 frames.

**Figure 15:**
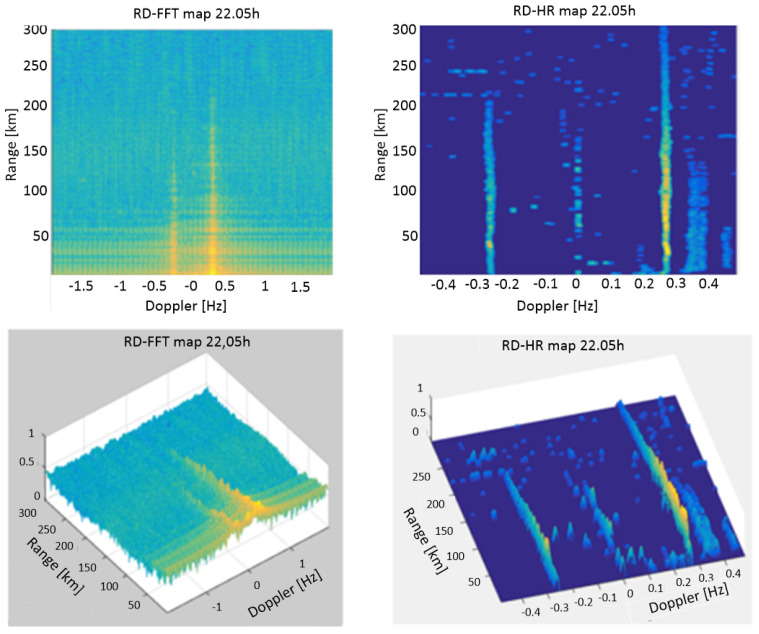
The comparison of RD-FFT map (**left**) and RD-HR map obtained by the proposed algorithm (**right**).

First step in the proposed algorithm is forming of High Resolution Range–Doppler map. We used uniform sampling method. Here, we can see properties of Range–Doppler maps and their main advantages and differences. In Figure 15 a comparative view of the RD-FFT map, obtained by the FFT algorithm, and a high-resolution RD-HR map, obtained by the new high-resolution algorithm, was presented. Both maps were calculated for an integration period of 256 frames with a duration of 0.260022 s each (integration interval of 66.5656 s). At this point, it is worth noting the difference between two notions related to the performance of a position estimation algorithm. The first one is the *target resolvability*, which quantifies the ability of the algorithm to perceive two targets close to each other as separate targets and not a single larger target, depending on the distance between them. The second notion is the *grid resolution*, which represents the density of the points at which the criterion function of the algorithm is calculated. Even though it can be chosen arbitrarily, care must be taken not to make it too coarse, otherwise the accuracy and target resolvability of the algorithm would be degraded. On the other hand, choosing the grid resolution to be too fine directly increases the computational complexity of the algorithm. The resolution of the RD-FFT map according to Doppler is basically determined by the resolution properties of the Fourier transform and in this case it is 0.0150 Hz. The range of Doppler frequencies obtained by the FFT algorithm is from −1.9229 Hz to +1.9229 Hz. The RD-HR map is calculated using a new high-resolution algorithm when, and unlike the FFT algorithm, there is a possibility to choose the bandwidth and grid resolution with which the RD-HR map is calculated. In this particular case, the RD-HR map for the integration period of 256 frames was calculated in the range −0.4804 Hz to +0.4804 Hz with a grid resolution of 0.0019 Hz (RD-HR map is calculated in 513 points as opposed to RD-FFT map calculated in 256 points). It follows from the above that the grid resolution of the RD-HR Doppler map is 7.8947 times better than Doppler resolution of the RD-FFT map. The range in which the RD-HR map is calculated from −0.4804 Hz to +0.4804 Hz is chosen so that in this case it includes the Doppler frequencies of ships of interest, including Bragg’s lines. This range can be set arbitrarily. The calculation of the RD-HR map was performed with a range grid resolution of 375 m. It is 4 times better than the range resolution used in the calculation of the RD-FFT map (1.5 km).

Comparing the RD-FFT and the new RD-HR map clearly shows the advantages of the RD-HR map in terms of target resolvability in Doppler domain, map contrast and the ability to detect targets. It was also noticed that successive detections of ships of interest are chained and that there is an overlap of contours of criterion functions of successive detections, which is the basis for formulating criteria for detection consistency.

RD-HR maps are formed for each antenna individually and independently. Target detection is performed on an averaged RD-HR map. A 5×5 Gaussian kernel function was chosen based on the analysis of the shape of the lobes of the RD-HR map in order to improve detection performance. In the second step, the azimuth is estimated using a high-resolution MUSIC type algorithm that is executed only for all detections in the RD-HR map. Because of that, numerical complexity and the time of algorithm execution is reduced. Angle grid resolution had chosen to be 0.2 degrees, and it is much better resolution then the resolution used in many algorithms which are currently in use (typically 1 degree). In monostatic radars of this type, the accuracy in the azimuth dimension is usually the lowest of all the three dimensions. Because of this, it is significant to improve the azimuth estimation accuracy, if the estimator and the useful information embedded in the received signals allow, as long as this increase in the accuracy does not increase the numerical complexity so much that the algorithm can no longer run in real-time. In this particular case, the increase in the numerical complexity of the *entire* algorithm when the azimuth grid is refined from $0.2^{\circ }$ to $0.1^{\circ }$ is negligible. There are a few more reasons why it is important to choose a finer azimuth resolution. Firstly, the lobes in the MUSIC-based criterion function are very narrow, so the grid resolution should be chosen such that there are enough points on each lobe to enable us to recover the location of its maximum correctly. Furthermore, the MUSIC-based algorithm has high target resolvability, which is better than the FFT resolution cell. This allows us to detect vessels at the same range and radial speed, but at slightly different azimuths (within one FFT resolution cell), but this can be done only if the azimuth grid resolution is fine enough.Also, refining the grid without bound will not improve the performance of the estimator arbitrarily, but, instead, the performance improvement would experience a saturation effect. This is because the amount of useful information in the raw signals is limited, and thus determines the performance bound. However, by refining the resolution in this case from $0.2^{\circ }$ to $0.1^{\circ }$ does increase the performance noticeably (the saturation still does not occur).

**Figure 16:**
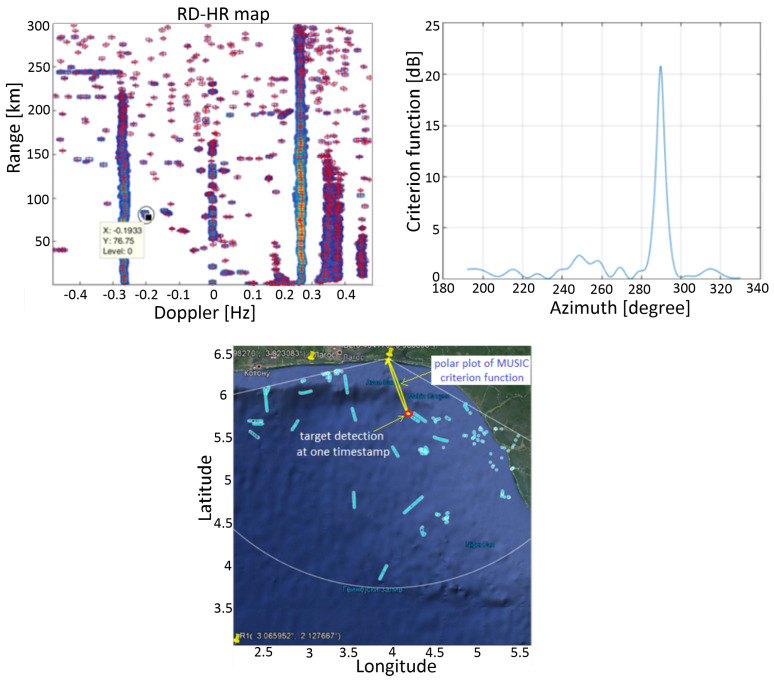
Detection of a vessel using the proposed algorithm: Detections in RD-HR map denoted by “+” markers (**upper left**), Azimuth estimation (A-HR criterion function) for the selected vessel detected in RD-HR map (**upper right**) and display of that detection (red circle) on a geographical map (**bottom**) with AIS data as a benchmark (light blue tracks), where the A-HR function is drawn as a yellow line (in polar coordinates, with the origin at the radar station) and scaled so that its maximum is at the position of the detection.

Figure 16 shows complete detection process.

**Figure 17:**
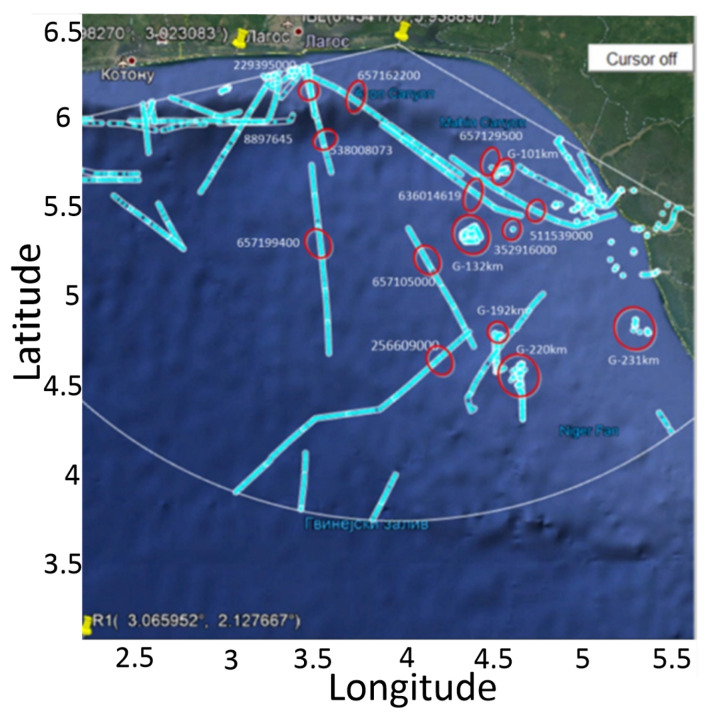
The complete AIS data in a time interval of approximately 5 h and randomly selected 10 vessels with their MMSI identifiers and 5 stationary located groups of vessels with G identifiers.

As mentioned earlier, in a time interval of approximately 5 h, we want to detect vessels using the proposed algorithm, and then compare the results with AIS data. Because of that, we made an experiment by randomly selected 10 vessels and monitored the detections throughout the time interval. The complete AIS data in a time interval of approximately 5 h is plotted in Figure 17.

**Figure 18:**
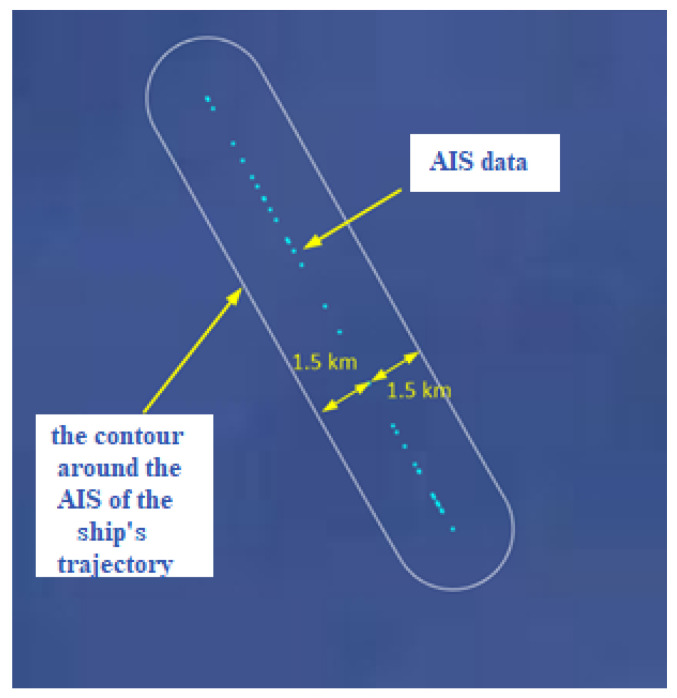
A contour formed around the AIS data.

First, a contour is formed around the AIS data, where the width of the contour is equal to the size of the initial resolution cell of 1.5 km. Then the criterion was made so that the detections and AIS data are monitored for one hour, hour by hour. According to this criterion, if the detection is within the contour we will consider that the real detection and not a false alarm. Figure 18 illustrate the forming of the criterion contour.

**Figure 19:**
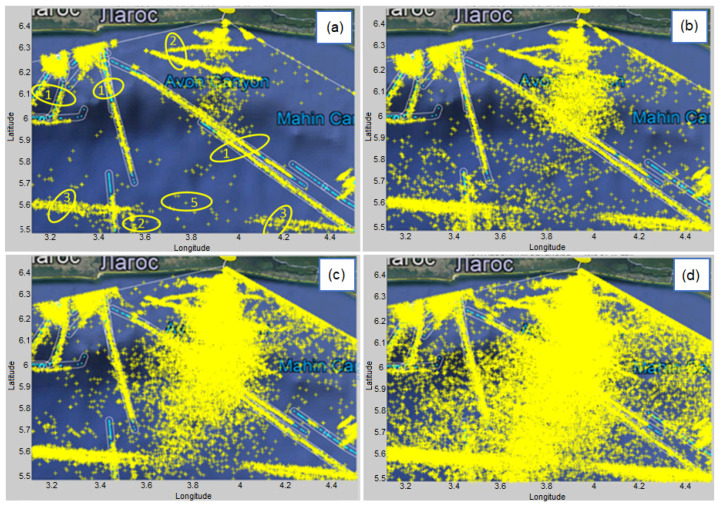
The display of all vessel’s detections (yellow markers) in a small part of the area, with AIS data as a benchmark (light blue tracks) in a time interval of 5 h for different detection parameters of the proposed algorithm: (**a**) K=5 and normalized threshold = 0.2 (**b**) K=5 and normalized threshold = 0.1 (**c**) K=10 and normalized threshold = 0.2 (**d**) K=10 and normalized threshold = 0.1.

We made an experiment by randomly selected 10 vessels whose basic information is available on websites (accessed date: 10 January 2022): www.vesseltracker.com, https://www.myshiptracking.com, https://www.marinetraffic.com and https://maritimeoptima.com. We will monitor the vessels by MMSI (A Maritime Mobile Service Identity) number. MMSI is a nine-digit number that uniquely identify vessel stations and it is sent over a radio frequency channel. Figure 19 shows all vessel’s detections in a time interval of 5 h for different detection parameters of the proposed algorithm. It can be clearly seen that as the order of the model *K* increases, the number of detections increases too, which is an expected result. Also with increasing the value of the normalized detection threshold, the number of detections decreases, which is a consequence of poorer detection of peaks in the Range–Doppler map.

Figure 19a also shows the markings of some individual tracks because we want to explain the nature of these tracks. Note that a small part of the area in Figure 16 was zoomed in and shown in Figure 19 so that the details, especially the categorization of the detections, could be clearly seen. Tracks marked by 1 indicate the traces for which AIS data exists. Light blue tracks represent the AIS data and we use this as the benchmark (as real vessel trajectories), where available. Note that AIS tracks are partially visible because they are overlayed (covered) by the target detections (yellow markers). The tracks marked by 2 indicate traces for which there is no AIS data, but from the aspect of the detection algorithm, this vessel can be considered as detectable. We know that these tracks correspond to real vessels, because this was confirmed by the tracker that was executed after the proposed detection algorithm (the tracker is outside the scope of the paper). The track marked with the number 3 is a consequence of ionospheric interference. Other scattered detections, marked with the number 5, are a consequence of the sea clutter. Note that the first-order Bragg’s lines in Figure 12 were suppressed in preprocessing, so that they do not appear in Figure 19. Additionally, even though Bragg’s lines are (more or less) concentrated in two regions on an RD-HR map, they appear dispersed on a geographical map. What can be noticed is that in all cases the detections match the AIS data very well, and in the next step an accuracy analysis will be made depending on the selected algorithm parameters (K and threshold).

**Figure 20:**
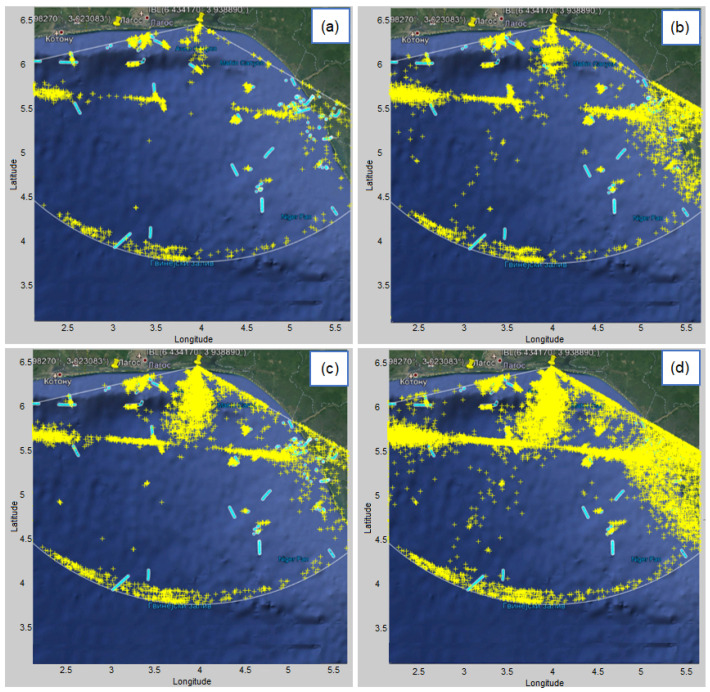
The display of all vessel’s detections (yellow markers) with AIS data as a benchmark (light blue tracks) in a time interval of 1 h (17–18 pm) for different detection parameters of the proposed algorithm: (**a**) K=5 and normalized threshold = 0.2 (**b**) K=5 and normalized threshold = 0.1 (**c**) K=10 and normalized threshold = 0.2 (**d**) K=10 and normalized threshold = 0.1.

Since we observe detections hour by hour, Figure 20 shows an example of the appearance of detections on a geographic map from 17 pm to 18 pm for different values of algorithm parameters. Graphs of this type are very useful and can be used to monitor changes by the hour, for example if there is a change in climatic conditions, or if there is a change from day to night, etc.

Note that the detections in Figure 20 were intentionally shown only for the interval 17:33–18:00, whereas the AIS data was shown for the entire hour (17:00–18:00), so that the beginnings (roughly the first 50%) of the AIS tracks would be visible (not overlayed by detections).

**Figure 21:**
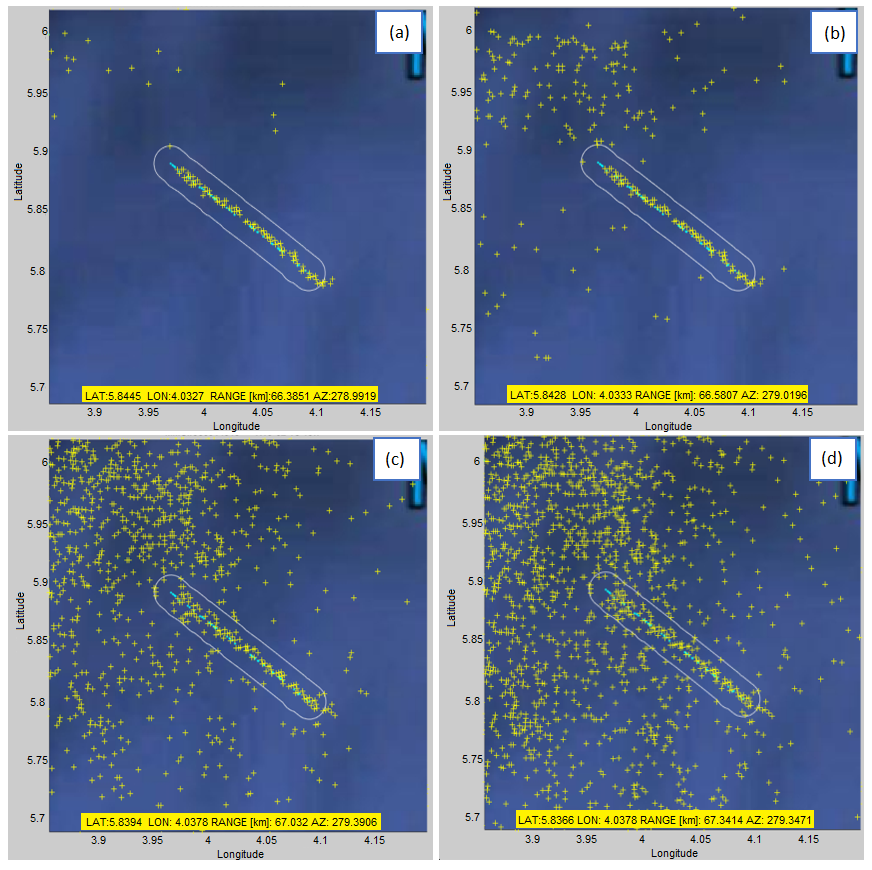
The display of all detections of vessel with MMSI = 636014619 (yellow markers) with AIS data as a benchmark (light blue tracks) in a time interval of 1 h (18–19 h) for different detection parameters of the proposed algorithm: (**a**) K=5 and normalized threshold = 0.2 (**b**) K=5 and normalized threshold = 0.1 (**c**) K=10 and normalized threshold = 0.2 (**d**) K=10 and normalized threshold = 0.1.

In the following analyses, the results of the detection of arbitrarily selected vessels will be presented in order to see the impact of certain parameters. Figure 21 shows detections for a vessel with MMSI = 636014619 in a time interval of 1 h (18–19 h) for different detection parameters of the proposed algorithm. Note that there is only one vessel in the selected area—the one inside the vessel contour (this was verified by the tracker). Tracks can be clearly seen and detections successfully follow the AIS data.

**Figure 22:**
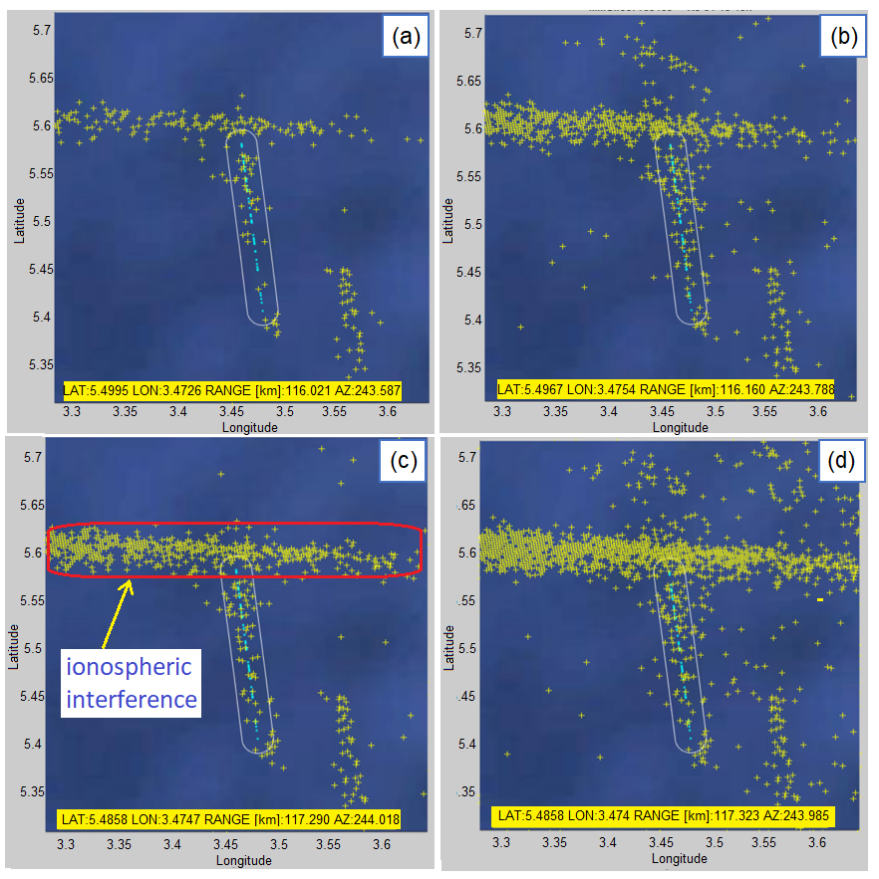
The display of all detections of vessel with MMSI = 657199400 (yellow markers) with AIS data as a benchmark (light blue tracks) in a time interval of 1 h (18–19 h) for different detection parameters of the proposed algorithm: (**a**) K=5 and normalized threshold = 0.2 (**b**) K=5 and normalized threshold = 0.1 (**c**) K=10 and normalized threshold = 0.2 (**d**) K=10 and normalized threshold = 0.1.

The highest number of detections is in the case when the model number is higher and the detection threshold is lower. This certainly increases the detectability of vessels, but also increases the number of false alarms. Figure 22 shows detections for a vessel with MMSI = 657,199,400 in a time interval of 1 h (18–19 h) for different detection parameters of the proposed algorithm when ionospheric interference is present. It interferes with the detection process but the tracks are still visible and the vessel is detectable for the whole period of time. Similarly, there is only one vessel in the selected area (verified by the tracker) and there is also strong ionospheric interference.

**Figure 23:**
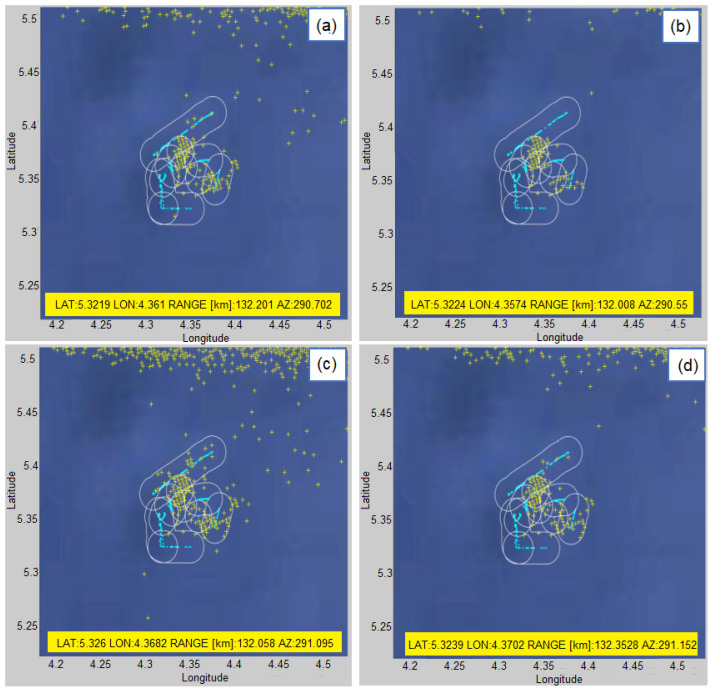
The display of all detections of groups of vessels with with G-123km identifier (yellow markers) with AIS data as a benchmark (light blue tracks) in a time interval of 1 h (18–19 h) for different detection parameters of the proposed algorithm: (**a**) K=5 and normalized threshold = 0.2 (**b**) K=5 and normalized threshold = 0.1 (**c**) K=10 and normalized threshold = 0.2 (**d**) K=10 and normalized threshold = 0.1.

The following example which is shown in Figure 23 will show how stationary targets are detected. One of the stationary targets, which is also shown on the map, will be taken. This target is labeled as G-132km. The model number and the choice of the detection threshold do not significantly affect the detectability of such a target and it is detectable in all cases.

**Figure 24:**
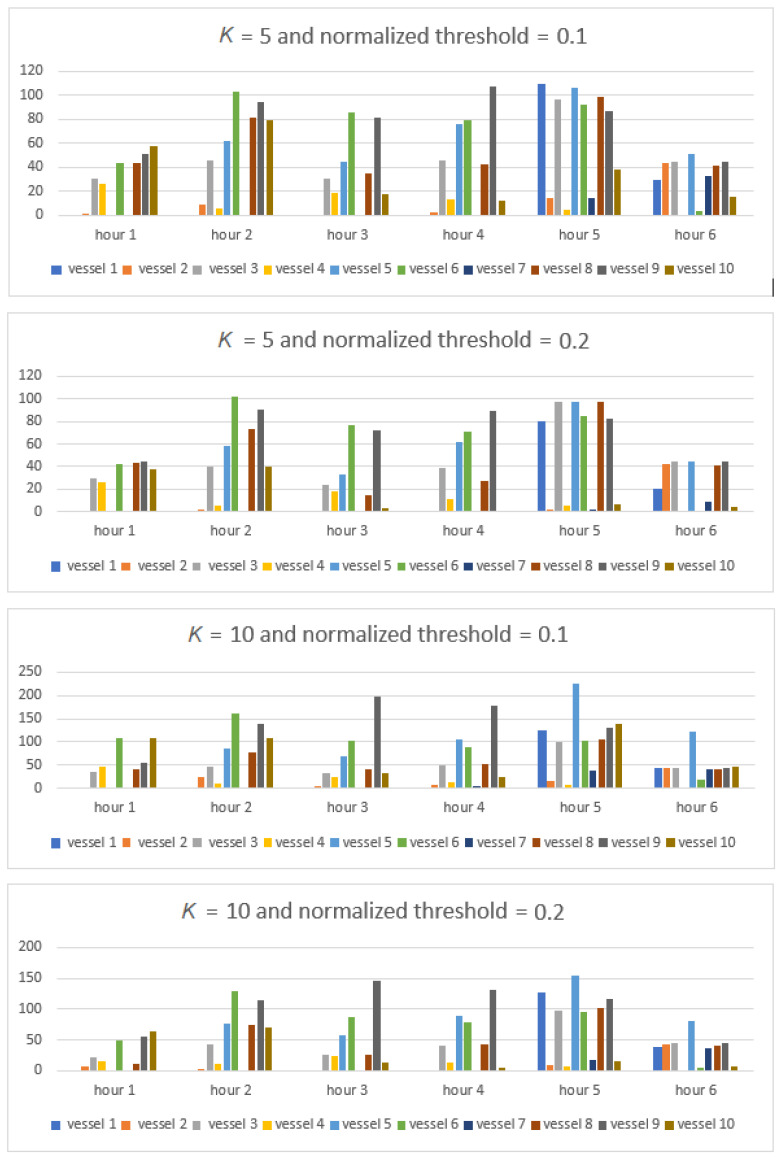
Number of vessel detections inside selected contours.

The following analyses will provide numerical data of the proposed algorithm for different parameters *K* and threshold in order to obtain their optimal values. Figure 24 shows the total number of detections inside the selected contour for all vessels. From this picture, you can clearly see which ships are detectable and in what period of time.

Based on all previous analyses, the assessment of vessel detectability was made. Table 1 and Table 2 show the final results.

**Figure 25:**
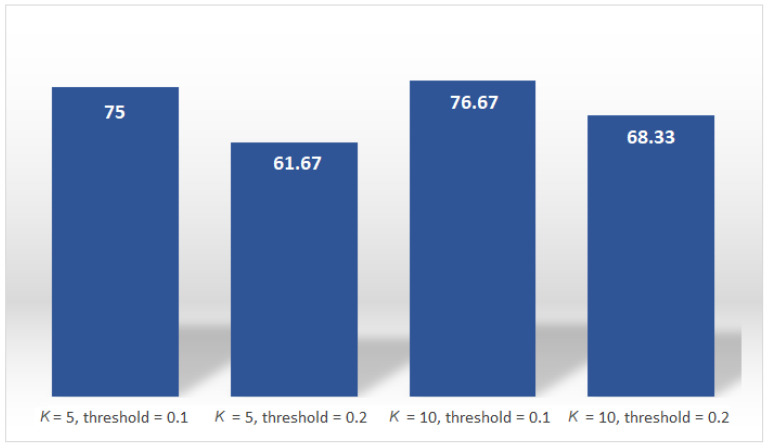
The percentage of detection success.

As can be seen from the previous figure, the detection success is high in all cases. In order to determine the optimal value of the algorithm parameters, in practical situations it is necessary to determine the percentage of detection success. Figure 25 shows the percentage of detection success of the proposed algorithm for different parameters *K* and threshold. In this case, the best performance is 76.67% for chosen algorithm parameters *K* = 10 and threshold 0.1. This clearly shows that the order of the model must be higher and the detection threshold lower in order for this percentage to be higher. But in addition to this, an important parameter can be the ratio of true detections and total number of detections, and it is desirable that this number be as small as possible so that there are not too many false alarms.

The ratio of total number of detections and number of detections within contours for all vessels and for all algorithm parameters is shown in Table 3.

To provide more useful information about the performance of the algorithm than the cumulative number of detections and false alarms inside vessel contours (Table 3), we approximately determined the probability of detection and the probability of a false alarm ($P_{d}$ and $P_{\mathrm{fa}}$). In order to determine $P_{d}$ and $P_{\mathrm{fa}}$, we made a criterion.

Suppose there is a single vessel in an area of interest in a given time interval. Let G be the set of points in the search grid (finite set). Also define $T=\left\{t_{1},t_{2},\ldots ,t_{n}\right\}$, as the set of timestamps in the given interval, A as the selected area of interest, B(t), t∈T as the ball centered at the benchmark location of the vessel at time *t*, and D(t) as the set of locations of the detections obtained by the algorithm from the segment with timestamp *t*. Then, we can define the area inside the contour around the vessel’s trajectory as

(34) $$C=\underset{t\in T}{⋃}B\left(t\right).$$

One possible estimate of the false alarm rate (FAR) is then

(35) $$P_{\mathrm{fa}}=\frac{\sum_{t\in T}\left|D(t)\backslash C\right|}{\left|\left(A\backslash C\right)\cap G\right|\cdot \left|T\right|},$$

where $\left|\cdot \right|$ denotes the number of elements of a set. Another way of defining the FAR estimate, which also counts the detections inside the contour, but outside the ball for a given *t* (it includes an extended area), is

(36) $$P_{\mathrm{fae}}=\frac{\sum_{t\in T}\left|D(t)\backslash B(t)\right|}{\sum_{t\in T}\left|\left(A\backslash B(t)\right)\cap G\right|}.$$

We also estimate the probability of detection, $P_{d}$, as the ratio of the number of timestamps *t* in which there is at least one detection inside the ball around the vessel, i.e., B(t)∩D(t)≠∅, and the total number of timestamps in which the vessel is present (in the given interval).

The ball is centered at the AIS location of the vessel at the given timestamp (the timestamp of the given signal segment) and its radius is equal to the size of the initial resolution cell of 1.5 km. Total number of time stamps in the selected period of time (18–19 h) is 105.

**Figure 26:**
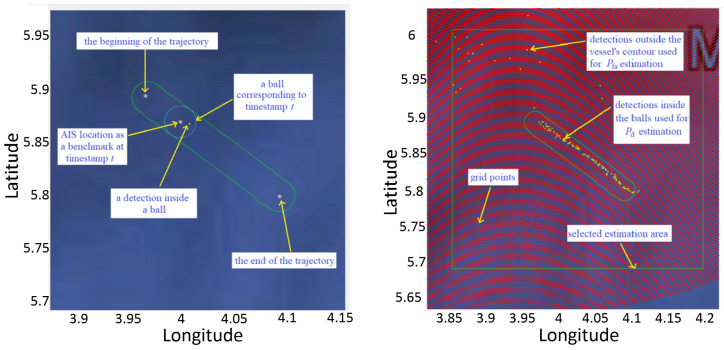
The forming of the ball (**left**) and the selection of the estimation area around the vessel’s contour (**right**).

Figure 26 shows the proposed criteria used to determine the probability of detection and the probability of false alarm.

Table 4 and Table 5 show the numerical results for two vessels presented in the paper. We have chosen these two vessels so that the detection of one is quite bothered by ionospheric interference, and the other is not. The results for the same vessels are presented in the previous part of the paper. The selected estimation area is the same as the area shown in the paper. In the cases in which the sample was too small to have at least 100 events of each of the two types (presence/absence of detection), we gave the estimate of the probability in the form of the ratio of the number of events of the given type and the total number of events (the sample size). In those cases, the probability estimate is not considered stable enough, but still provides some useful information about the behavior of the algorithm.

Increasing *K* from 5 to 10 for the vessel in Table 4 increases $P_{d}$ by 0.1143 and 0.0286 for threshold values of 0.1 and 0.2, respectively, while the FAR remains very low (in the order of $10^{-4}$). Similarly, for the vessel in Table 5 the increase in $P_{d}$ is 0.0762 and 0.1143.

In addition, one of the most important advantages of the high-resolution algorithm presented in this paper is target resolvability.

As it is well known, target resolvability in HFSWR is limited by the size of Range–Doppler-azimuth cells in 3D RDA map. The size of Range–Doppler-azimuth cell provided by DFT based primary signal processing (usually applied in HFSWR) is primarily limited by the resolution properties of DFT (the number of samples in range and Doppler domain and the number of antennas in linear antenna array). In the paper we proposed MUSIC-based methods for high-resolution Doppler and azimuth estimation. It is also known that high-resolution methods can resolve signal targets which are inside Rayleigh resolution bandwidth (in this case in Doppler and azimuth domain). So, it is clear that the size of Range–Doppler-azimuth cells provided by high-resolution methods are smaller then those provided by DFT methods. It means that the application of high-resolution methods in HFSWR can improve its target resolvability. Practically, it means, for example, that two targets at the same range and Doppler (this scenario is possible in practice) can be resolved in spatial (azimuth) domain.

In the presented signal scenario, we did not have two close targets representative for the illustration of better resolvability. So, we found a representative close target scenario from many other scenarios for which we have acquired signals.

**Figure 27:**
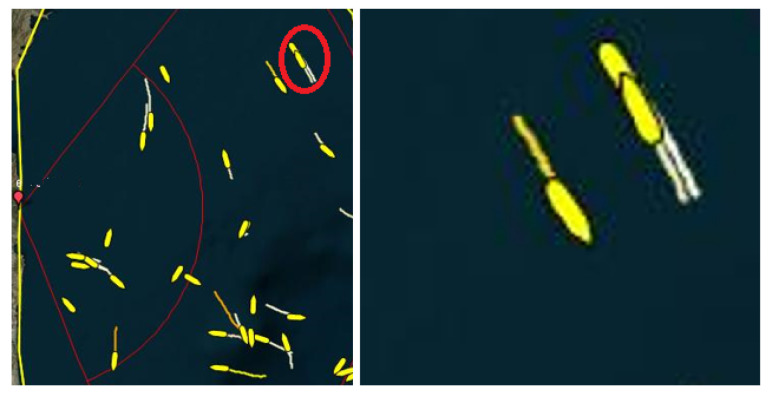
The display of two vessels (rounded in red) that were very close to each other (**left**) and zoomed display of these vessels (**right**).

In Figure 27, we show an example with two vessels that were very close to each other. According to the detections shown in the figure, the algorithm has a high target resolvability rate, even for vessels at the same range and Doppler shift.

And what is very important, the proposed high-resolution algorithm presented in the paper achieves real-time processing. The selected chirp duration is 0.260022 s, and since we want to output the results after every 128 frames, the real-time requirement is 33.28 s. Thus, the processing time of one segment must be shorter than 33.28 s. Also, the parallelization of the program was made, so that it is executed on several CPU cores and runs on on a PC computer with i7 CPU, and on a better CPU (AMD Ryzen) in real time. It should be emphasized that the parallelization was done for the most complex part of the algorithm (calculation of the RD-HR map) where CPU utilization is 100%, while in other less computationally demanding parts it is not. Table 6 shows the average segment processing time for a sample of 200 segments.

**Figure 28:**
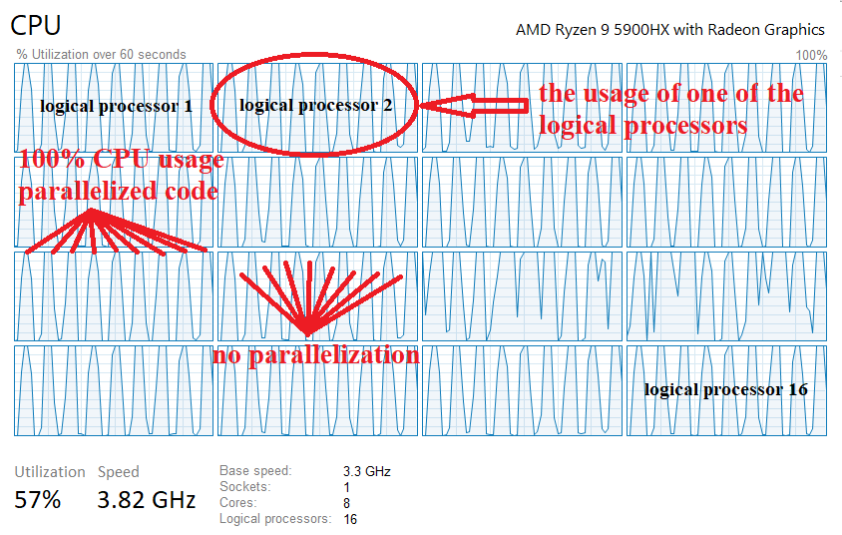
Logical processors usage in multithread software.

Figure 28 shows logical processors usage in the multithread software.

## 5. Conclusions

In this paper, we presented new developed algorithm for FFT-based range and MUSIC-based high-resolution Doppler and Azimuth estimation in HFSWRs. The performance of the proposed algorithm based on experimental study are also presented. We analyzed numerical results of the proposed algorithm for different parameters *K* and threshold values in order to obtain their optimal values. Based on the analyses, the assessment of vessel detectability was made. We improved the detectability in the selected time interval.The highest number of detections is in the case when the model number is higher and the detection threshold is lower, and what is more important, the ratio of total number of detections and true detections is not too high, and the percentage of detection success is high. Based on the experimental results, the assessment of vessel detectability was made. The properties of RD-HR maps are presented and also their main advantages and differences, related to RD-FFT maps, in order to have better target detectability. An RD-HR map has a higher contrast and it is more suitable for use in HFSWRs then RD-FFT maps. The contribution is also the compensation of the Doppler shift before high-resolution azimuth estimation which is performed using a high-resolution MUSIC-type algorithm, that is executed for each detection in the RD-HR map. Because of that, numerical complexity and the algorithm execution time are reduced. We do not need to process all the points from the RD-HR map, but only detections. We also form the criterion to compare detections and AIS data. It can be noticed that in all cases the detections match the AIS data very well, and this comparison can be very useful to verify empirically obtained results. Tracks can be clearly seen and detections successfully follow the AIS data.

We have also made rough estimates of the probability of detection and the probability of a false alarm, which show the advantages of the proposed algorithm.

In HFSWRs, CFAR detectors are usually used. The detection algorithm that we proposed, which comes from the field of Image processing, eliminates spurious single-pixel peaks in the criterion function and it can improve detectability by adapting the shape of the kernel to the shape of the lobes in the RD-HR map.

The high-resolution MUSIC-based algorithm was proposed for both the RD-HR map and the azimuth estimation. It increases the target resolvability of the radar both in Doppler and azimuth domain, as well as the detectability of the targets. The MUSIC-based algorithm was improved by using non-uniform sampling across the signal frames (in Doppler domain), to drastically reduce numerical complexity, while sacrificing little performance.

These results are obtained in a real environment within an experimental study. Therefore, the algorithm may have practical application in the future and have great potential because the surveillance with HFSWR radar can be done continuously, 24 h a day, 7 days a week at the significantly lower costs. It is also possible to define potentially suspicious activities and immediately warn the user about it. An extremely large detection range is therefore possible without the so-called “Blind zones”. Also, high reliability is enabled and possible integration with an automatic identification system (AIS) that provides the information about ships that are currently in a zone of interest. Therefore, all of the above represent a challenge to many researchers around the world to further develop and define HFSWR radar algorithms.

A special challenge is to compare the performance of new algorithms with existing algorithms for primary signal processing, as well as to define mathematical models that will lead to better ship detectability and better radar resolution properties, as well as the ability to detect some ships that are not detectable or separable by distance/azimuth using the currently used algorithms for primary signal processing.

The algorithm is potentially applicable for other purposes, for example for surveillance and other targets of interest, such as detection of sea currents, sea winds, icebergs, and can also be used to search and rescue people in the case the ship is lost from AIS and if an accident occurs, in the fishing, in the exploitation of marine resources, as well as tsunami detection, which can lead to saving many human lives, etc.

As for the future research directions, it will be important to explore a few key points that could improve the proposed method in practical situations further:

- Mathematical modeling and the simulation of the whole system, which includes the setting of all the parameters of the multi-target signal scenario (such as interference, propagation, see clutter and signal parameters, such as SNR and RCS). That way we would also be able to estimate the false alarm rate and the probability of detection in a fully controlable scenario.
- Automatic selection of detection thresholds dependent of the distance or weather conditions, sea clutter and external interference, that is better than the experimentally obtained threshold in terms of detection results;
- Suitable algorithm used to estimate parameter *K*, such as an adaptive determination method, or some custom variant. As we noticed, AIC and MDL give a higher value of *K* than the actual value;
- Automatic segment length selection;
- Algorithmic optimization for obtaining non-uniform frame selection based on which the RD map is calculated, which leads to a lower numerical complexity.

Most known technical radar solutions such as WERA, ONERA, OSMAR use the same type of primary signal processing based on Fourier spectral analysis. In all the listed HFSWRs, in the first step, by applying the Fourier transform, an RD map is formed. Target detection is performed on an RD map using one or more CFAR detector variants. In the second step, the direction estimate is determined for the targets detected in the RD map using the classic single snapshot beamformer.

High-resolution methods represent a new solution in relation to the state-of-the-art in the field of signal processing in HFSWRs and certainly lead to the improved detection and separability of close targets.

## Figures and Tables

**Table 1:** Detection results for K=5 and different threshold values in the selected period of time.

Detection Parameter *K* = 5													
Vessel Number	Vessel ID	Threshold = 0.1						Threshold = 0.2					
		17 h	18 h	19 h	20 h	21 h	22 h	17 h	18 h	19 h	20 h	21 h	22 h
1	229395000	✕	✕	✕	✕	✓	✓	✕	✕	✕	✕	✓	✓
2	256609000	✕	✕	✕	✕	✓	✓	✕	✕	✕	✕	✕	✓
3	352916000	✓	✓	✓	✓	✓	✓	✓	✓	✓	✓	✓	✓
4	511539000	✓	✕	✓	✓	✕	✕	✓	✕	✓	✓	✕	✕
5	358008073	✕	✓	✓	✓	✓	✓	✕	✓	✓	✓	✓	✓
6	636014619	✓	✓	✓	✓	✓	✓	✓	✓	✓	✓	✓	✕
7	352915000	✓	✓	✓	✓	✓	✓	✓	✓	✓	✓	✓	✓
8	657105000	✕	✕	✕	✕	✓	✓	✕	✕	✕	✕	✕	✕
9	657162200	✓	✓	✓	✓	✓	✓	✓	✓	✓	✓	✓	✓
10	657199400	✓	✓	✓	✓	✓	✓	✓	✓	✕	✕	✕	✕

✓—detected, ✕—not detected or no AIS.

**Table 2:** Detection results for K=10 and different threshold values in the selected period of time.

Detection Parameter *K* = 10													
Vessel Number	Vessel ID	Threshold = 0.1						Threshold = 0.2					
		17 h	18 h	19 h	20 h	21 h	22 h	17 h	18 h	19 h	20 h	21 h	22 h
1	229395000	✕	✕	✕	✕	✓	✓	✕	✕	✕	✕	✓	✓
2	256609000	✕	✓	✕	✓	✕	✓	✕	✕	✕	✕	✕	✓
3	352916000	✓	✓	✓	✓	✓	✓	✓	✓	✓	✓	✓	✓
4	511539000	✓	✓	✓	✓	✕	✕	✓	✓	✓	✓	✕	✕
5	358008073	✕	✓	✓	✓	✓	✓	✕	✓	✓	✓	✓	✓
6	636014619	✓	✓	✓	✓	✓	✓	✓	✓	✓	✓	✓	✕
7	657129500	✓	✓	✓	✓	✓	✓	✓	✓	✓	✓	✓	✓
8	657105000	✕	✕	✕	✕	✓	✓	✕	✕	✕	✕	✓	✓
9	657162200	✓	✓	✓	✓	✓	✓	✓	✓	✓	✓	✓	✓
10	657199400	✓	✓	✓	✓	✓	✓	✓	✓	✓	✕	✓	✕

✓—detected, ✕—not detected or no AIS.

**Table 3:** The ratio of total number of detections and detections within contours for all vessels.

Algorithm Parameters		Total Number of Detections within Contours for All Vessels		Total Number of Non-Contour Detections for All Vessels		The Ratio of Total Number of Detections and Number of Detections within Contours for All Vessels	
		Threshold 0.1	Threshold 0.2	Threshold 0.1	Threshold 0.2	Threshold 0.1	Threshold 0.2
*K* = 5	17 h	976	780	4856	1294	5.97	2.66
	18 h	1600	1315	18435	6849	12.52	6.21
	19 h	952	669	6150	1400	7.46	3.09
	20 h	1097	910	5295	1170	5.83	2.28
	21 h	1968	1685	6024	1397	4.06	1.83
	22 h	954	804	2426	622	3.54	1.77
*K* = 10	17 h	1390	1016	13298	5093	10.57	6.01
	18 h	2091	1634	39678	18136	19.97	12.10
	19 h	1363	869	18264	5878	14.40	7.76
	20 h	1371	1052	17811	4946	13.99	5.70
	21 h	2578	2031	21154	7191	9.20	4.54
	22 h	1267	1008	8839	3254	7.98	4.23

**Table 4:** $P_{d}$ and $P_{f}$ of the vessel with MMSI = 636014619 in a time interval of 1 h (18–19 h).

Detection Parameters	$P_{d}$	$P_{\mathrm{fa}}$	$P_{\mathrm{fae}}$
*K* = 5, threshold = 0.2	0.7238	21/1,689,555 *	48/1,759,737 *
*K* = 5, threshold = 0.1	0.7238	1.0417 $\times 10^{-4}$	1.1536 $\times 10^{-4}$
*K* = 10, threshold = 0.2	0.7524	4.5278 $\times 10^{-4}$	4.6257 $\times 10^{-4}$
*K* = 10, threshold = 0.1	0.8381	7.6470 $\times 10^{-4}$	7.7625 $\times 10^{-4}$

* too small a sample to estimate.

**Table 5:** $P_{d}$ and $P_{f}$ of the vessel with MMSI = 657199400 in a time interval of 1 h (18–19 h).

Detection Parameters	$P_{d}$	$P_{\mathrm{fa}}$	$P_{\mathrm{fae}}$
*K* = 5, threshold = 0.2	0.2095	1.7061 $\times 10^{-4}$	1.7565 $\times 10^{-4}$
*K* = 5, threshold = 0.1	0.2952	5.6351 $\times 10^{-4}$	5.7442 $\times 10^{-4}$
*K* = 10, threshold = 0.2	0.3238	5.3808 $\times 10^{-4}$	5.4040 $\times 10^{-4}$
*K* = 10, threshold = 0.1	0.3714	13.3301 $\times 10^{-4}$	13.3301 $\times 10^{-4}$

**Table 6:** The average segment processing time obtained by the implemented detection software.

CPU Type	Processing Time (1-Thread Software)	Processing Time (Multi-Thread Software)
Intel CORE i7 1075H	32.2 s	8.1 s
AMD Ryzen 9 5900HX	22.9 s	4.4 s

## Data Availability

Not applicable.

## Abbreviations

The following abbreviations are used in this manuscript:

HFSWR	High Frequency Surface Wave Radar
FFT	Fast Fourier Transform
OTHR	Over The Horizon Radar
EEZ	Exclusive Economic Zone
DoA	Direction of Arrival
MUSIC	Multiple Signal Classification
A/D	Analog to Digital
AIS	Automatic Identification System
HR	High Resolution
RF	Radio Frequency
MMSI	Maritime Mobile Service Identity
RD	Range–Doppler

## Appendix A. Derivation of Dechirped Signal

Complete derivations of equations for the dechirped signal are given here.

(A1) $$t-\tau_{n}^{\left(q\right)}\left(\tilde{t}\right)=t-\frac{2R_{m}^{\left(q\right)}}{c}-\frac{2v_{m}^{\left(q\right)}}{c}t-\tau_{An}^{\left(q\right)}$$

(A2) $${\left(t-\tau_{n}^{\left(q\right)}\left(\tilde{t}\right)\right)}^{2}=t^{2}-2t\tau_{n}^{\left(q\right)}\left(\tilde{t}\right)+{\left(\tau_{n}^{\left(q\right)}\left(\tilde{t}\right)\right)}^{2}$$

(A3) $$\begin{array}{cc} \; & {\left(\tau_{n}^{\left(q\right)}\left(\tilde{t}\right)\right)}^{2}={\left(\frac{2}{c}\left(R_{m}^{\left(q\right)}+v_{m}^{\left(q\right)}t\right)+\tau_{An}^{\left(q\right)}\right)}^{2}\\ \; & =\frac{4}{c^{2}}{\left(R_{m}^{\left(q\right)}+v_{m}^{\left(q\right)}t\right)}^{2}+\frac{4}{c}\left(R_{m}^{\left(q\right)}+v_{m}^{\left(q\right)}t\right)\tau_{An}^{\left(q\right)}+{\left(\tau_{An}^{\left(q\right)}\right)}^{2}\\ \; & =\frac{4}{c^{2}}\left({\left(R_{m}^{\left(q\right)}\right)}^{2}+2R_{m}^{\left(q\right)}v_{m}^{\left(q\right)}t+{\left(v_{m}^{\left(q\right)}\right)}^{2}t^{2}\right)+\frac{4}{c}R_{m}^{\left(q\right)}\tau_{An}^{\left(q\right)}+\frac{4}{c}v_{m}^{\left(q\right)}t\tau_{An}^{\left(q\right)}+{\left(\tau_{An}^{\left(q\right)}\right)}^{2}\\ \; & =\frac{4}{c^{2}}{\left(R_{m}^{\left(q\right)}\right)}^{2}+\frac{8}{c^{2}}R_{m}^{\left(q\right)}v_{m}^{\left(q\right)}t+\frac{4}{c^{2}}{\left(v_{m}^{\left(q\right)}\right)}^{2}t^{2}+\frac{4}{c}R_{m}^{\left(q\right)}\tau_{An}^{\left(q\right)}+\frac{4}{c}v_{m}^{\left(q\right)}t\tau_{An}^{\left(q\right)}+{\left(\tau_{An}^{\left(q\right)}\right)}^{2}. \end{array}$$

In the first case, for $\tau_{n}^{\left(q\right)}\left(\tilde{t}\right)<t<T$, the dechirped signal is

(A4) $$\begin{array}{cc} \; & x_{n}^{\left(q\right)}\left(\tilde{t}\right)r{\left(\tilde{t}\right)}^{*}=a^{\left(q\right)}r\left(t-\tau_{n}^{\left(q\right)}\left(\tilde{t}\right)\right)r{\left(t\right)}^{*}\\ \; & =a^{\left(q\right)}e^{j2\pi \left(\left(f_{c}-\frac{B}{2}\right)\left(t-\tau_{n}^{\left(q\right)}\left(\tilde{t}\right)\right)+\frac{B}{2T}{\left(t-\tau_{n}^{\left(q\right)}\left(\tilde{t}\right)\right)}^{2}\right)}e^{-j2\pi \left(\left(f_{c}-\frac{B}{2}\right)t+\frac{B}{2T}t^{2}\right)}\\ \; & =a^{\left(q\right)}e^{j2\pi \left(\left(f_{c}-\frac{B}{2}\right)\left(t-\tau_{n}^{\left(q\right)}\left(\tilde{t}\right)\right)+\frac{B}{2T}{\left(t-\tau_{n}^{\left(q\right)}\left(\tilde{t}\right)\right)}^{2}-\left(f_{c}-\frac{B}{2}\right)t-\frac{B}{2T}t^{2}\right)}=a^{\left(q\right)}e^{j2\pi \varphi_{m,n}^{\left(q\right)}\left(t\right)}. \end{array}$$

Thus, the phase term $\varphi_{m,n}^{\left(q\right)}\left(t\right)$ in the previous equation can be derived as follows:

(A5) φm,n(q)(t)=fct−Bt2−fcτn(q)(t˜)+B2τn(q)(t˜)+B2Tt2−B2T2tτn(q)(t˜)+B2Tτn(q)(t˜)2−fct+Bt2−B2Tt2=τn(q)(t˜)−fc+B2−BtT+B2Tτn(q)(t˜)2=2Rm(q)c+2vm(q)ct+τAn(q)−fc+B2−BtT+B2Tτn(q)(t˜)2=−2fcRm(q)c−2fcvm(q)ct−fcτAn(q)+Rm(q)Bc+vm(q)Bct+B2τAn(q)−2Rm(q)BcTt−2vm(q)BcTt2−BTτAn(q)t+2BTc2Rm(q)2+4BTc2Rm(q)vm(q)t+2BTc2vm(q)2t2+2BTcRm(q)τAn(q)+2BTcvm(q)τAn(q)t+B2TτAn(q)2=t−2vm(q)cfc−B2−2BRm(q)cT+4BRm(q)vm(q)c2T+t2−2vm(q)BcT+2(vm(q))2Bc2T+tτAn(q)−BT+2Bvm(q)Tc+τAn(q)2B2T+2BTc2Rm(q)2−2Rm(q)cfc−B2+τAn(q)−fc+B2+2BRm(q)Tc.

Finally, for $\tau_{n}^{\left(q\right)}\left(\tilde{t}\right)<t<T$, dechirped signal can be expressed as:

(A6) $$\begin{array}{cc} x_{n}^{\left(q\right)}\left(\tilde{t}\right)r{\left(\tilde{t}\right)}^{*} & =a^{\left(q\right)}e^{j2\pi t\left(-\frac{v_{m}^{\left(q\right)}}{c}\left(2f_{c}-B\right)-\frac{2BR_{m}^{\left(q\right)}}{cT}+\frac{4BR_{m}^{\left(q\right)}v_{m}^{\left(q\right)}}{c^{2}T}\right)}\times e^{j2\pi t\tau_{An}^{\left(q\right)}\left(-\frac{B}{T}+\frac{2Bv_{m}^{\left(q\right)}}{Tc}\right)}\\ \; & \times e^{j2\pi t^{2}\left(-\frac{2v_{m}^{\left(q\right)}B}{cT}+\frac{2{\left(v_{m}^{\left(q\right)}\right)}^{2}B}{c^{2}T}\right)}\times e^{j2\pi \tau_{An}^{\left(q\right)}\left(-f_{c}+\frac{B}{2}+\frac{2BR_{m}^{\left(q\right)}}{cT}\right)}\\ \; & \times e^{j2\pi {\left(\tau_{An}^{\left(q\right)}\right)}^{2}\frac{B}{2T}}\times e^{j2\pi \left(\frac{2B{\left(R_{m}^{\left(q\right)}\right)}^{2}}{Tc^{2}}-\frac{R_{m}^{\left(q\right)}\left(2f_{c}-B\right)}{c}\right)}. \end{array}$$

In the second case, for $0<t<\tau_{n}^{\left(q\right)}\left(\tilde{t}\right)$, the dechirped signal is

(A7) $$\begin{array}{cc} \; & x_{n}^{\left(q\right)}\left(\tilde{t}\right)r{\left(\tilde{t}\right)}^{*}=a^{\left(q\right)}r\left(t-\tau_{n}^{\left(q\right)}\left(\tilde{t}\right)+T\right)r{\left(t\right)}^{*}\\ \; & =a^{\left(q\right)}e^{j2\pi \left(\left(f_{c}-\frac{B}{2}\right)\left(t-\tau_{n}^{\left(q\right)}\left(\tilde{t}\right)+T\right)+\frac{B}{2T}{\left(t-\tau_{n}^{\left(q\right)}\left(\tilde{t}\right)+T\right)}^{2}\right)}e^{-j2\pi \left(\left(f_{c}-\frac{B}{2}\right)t+\frac{B}{2T}t^{2}\right)}\\ \; & =a^{\left(q\right)}e^{j2\pi \left(\left(f_{c}-\frac{B}{2}\right)\left(t-\tau_{n}^{\left(q\right)}\left(\tilde{t}\right)+T\right)+\frac{B}{2T}{\left(t-\tau_{n}^{\left(q\right)}\left(\tilde{t}\right)+T\right)}^{2}-\left(f_{c}-\frac{B}{2}\right)t-\frac{B}{2T}t^{2}\right)}=a^{\left(q\right)}e^{j2\pi \varphi_{m,n}^{\left(q\right)}\left(t\right)}. \end{array}$$

In the previous equation, we found:

(A8) $${\left(t-\tau_{n}^{\left(q\right)}\left(\tilde{t}\right)+T\right)}^{2}=t^{2}-2t\tau_{n}^{\left(q\right)}\left(\tilde{t}\right)+{\left(\tau_{n}^{\left(q\right)}\left(\tilde{t}\right)\right)}^{2}+2tT-2T\tau_{n}^{\left(q\right)}\left(\tilde{t}\right)+T^{2}.$$

In the second case, the phase term $\varphi_{m,n}^{\left(q\right)}\left(t\right)$ from the first case has to be supplemented by following terms:

(A9) B2T2tT−2Tτn(q)(t˜)+T2+fcT−BT2=tB−2Bcvm(q)+BT2−2BcRm(q)+fcT−BT2−τAn(q)B.

Finally, in the second case, when $0<t<\tau_{n}^{\left(q\right)}\left(\tilde{t}\right)$, the dechirped signal is

(A10) $$\begin{array}{cc} x_{n}^{\left(q\right)}\left(\tilde{t}\right)r{\left(\tilde{t}\right)}^{*} & =a^{\left(q\right)}e^{j2\pi t\left(-\frac{v_{m}^{\left(q\right)}}{c}\left(2f_{c}+B\right)-\frac{2BR_{m}^{\left(q\right)}}{cT}+\frac{4BR_{m}^{\left(q\right)}v_{m}^{\left(q\right)}}{c^{2}T}+B\right)}\times e^{j2\pi t\tau_{An}^{\left(q\right)}\left(-\frac{B}{T}+\frac{2Bv_{m}^{\left(q\right)}}{Tc}\right)}\\ \; & \times e^{j2\pi t^{2}\left(-\frac{2v_{m}^{\left(q\right)}B}{cT}+\frac{2{\left(v_{m}^{\left(q\right)}\right)}^{2}B}{c^{2}T}\right)}\times e^{j2\pi \tau_{An}^{\left(q\right)}\left(-f_{c}-\frac{B}{2}+\frac{2BR_{m}^{\left(q\right)}}{cT}\right)}\\ \; & \times e^{j2\pi {\left(\tau_{An}^{\left(q\right)}\right)}^{2}\frac{B}{2T}}\times e^{j2\pi \left(\frac{2B{\left(R_{m}^{\left(q\right)}\right)}^{2}}{Tc^{2}}-\frac{R_{m}^{\left(q\right)}}{c}\left(2f_{c}+B\right)+f_{c}T\right)}. \end{array}$$

## Appendix B. Approximation of the Dechirped Signal

Under the assumption that $v_{m}^{\left(q\right)}=v^{\left(q\right)}=\mathrm{const}$ and that $v_{m}^{\left(q\right)}t$ is negligible (because we neglect Doppler effect during one frame), we have

(A11) $$\begin{array}{cc} R_{m}^{\left(q\right)} & \approx R_{0}^{\left(q\right)}+mTv^{\left(q\right)} \end{array}$$

(A12) $$\tau_{n}^{\left(q\right)}\left(\tilde{t}\right)\approx \tau_{m,n}^{\left(q\right)}=\frac{2R_{m}^{\left(q\right)}}{c}+\tau_{An}^{\left(q\right)}.$$

Also the terms containing $\frac{1}{c^{2}}$ are negligible, so we get an approximated model of the dechirped signal.

In the first case, for $\tau_{m,n}^{\left(q\right)}<t<T$, the phase term $\varphi_{m,n}^{\left(q\right)}\left(t\right)$ from the Equation (A5) can be derived as follows:

(A13) φm,n(q)(t)=−2tv(q)cfc−B20−2tBRm(q)cT+4tBRmqv(q)c2T0+tτAn(q)−BT+tτAn(q)2Bv(q)Tc0+t2−2v(q)BcT0+t22(v(q))2Bc2T0+τAn(q)−fc+B2+2BRm(q)cT+τAn(q)2B2T+2BTc2Rm(q)20−2Rm(q)cfc−B2=−2tBcTR0(q)+mTv(q)+tτAn(q)−BT+τAn(q)−fc+B2+τAn(q)2BcTR0(q)+mTv(q)+τAn(q)2B2T−2cfc−B2R0(q)+mTv(q)=R0(q)−2tBcT−2cfc−B2+v(q)−2tBcTmT+v(q)−2cfc−B2mT+τAn(q)−BtT−fc+B2+τAn(q)R0(q)2BcT+τAn(q)v(q)−2BcTmT+τAn(q)2B2T.

Finally, for $\tau_{m,n}^{\left(q\right)}<t<T$, the approximation of the dechirped signal can be expressed as:

(A14) $$\begin{array}{cc} y_{n}^{\left(q\right)}\left(\tilde{t}\right) & =a^{\left(q\right)}e^{j2\pi R_{0}^{\left(q\right)}\left(-\frac{2tB}{cT}-\frac{2f_{c}-B}{c}\right)}\times e^{j2\pi v^{\left(q\right)}\left(-\frac{2f_{c}-B}{c}mT-\frac{2mtB}{c}\right)}\\ \; & \times e^{j2\pi \tau_{An}^{\left(q\right)}\left(\frac{-Bt}{T}-f_{c}+\frac{B}{2}\right)}\times e^{j2\pi \tau_{An}^{\left(q\right)}R_{0}^{\left(q\right)}\frac{2B}{cT}}\\ \; & \times e^{j2\pi \tau_{An}^{\left(q\right)}v^{\left(q\right)}\frac{2Bm}{c}}\times e^{j2\pi {\left(\tau_{An}^{\left(q\right)}\right)}^{2}\frac{B}{2T}}. \end{array}$$

In the second case, for $0<t<\tau_{m,n}^{\left(q\right)}$, the phase term $\varphi_{m,n}^{\left(q\right)}\left(t\right)$ from the first case has to be supplemented by following terms:

(A15) tB−t2Bcv(q)0−2BcRm(q)+fcT−τAn(q)B==tB−2BcR0(q)+mTv(q)+fcT−τAn(q)B

So, for $0<t<\tau_{m,n}^{\left(q\right)}$, the approximation of the dechirped signal can be expressed as:

(A16) $$\begin{array}{cc} y_{n}^{\left(q\right)}\left(\tilde{t}\right) & =a^{\left(q\right)}e^{j2\pi R_{0}^{\left(q\right)}\left(-\frac{2tB}{cT}-\frac{2f_{c}+B}{c}\right)}\times e^{j2\pi v^{\left(q\right)}\left(-\frac{2f_{c}+B}{c}mT-\frac{2mtB}{c}\right)}\\ \; & \times e^{j2\pi \tau_{An}^{\left(q\right)}\left(\frac{-Bt}{T}-f_{c}-\frac{B}{2}\right)}\times e^{j2\pi \tau_{An}^{\left(q\right)}R_{0}^{\left(q\right)}\frac{2B}{cT}}\\ \; & \times e^{j2\pi \tau_{An}^{\left(q\right)}v^{\left(q\right)}\frac{2Bm}{c}}\times e^{j2\pi {\left(\tau_{An}^{\left(q\right)}\right)}^{2}\frac{B}{2T}}\times e^{j2\pi \left(Bt+f_{c}T\right)}. \end{array}$$

## References

[1] Headrick J.M., Skolnik M.I. (1974). Over-the-Horizon Radar in the HF Band. Proc. IEEE.

[2] Georges T.M., Harlan J.A. (1994). New horizons for over-the-horizon radar?. IEEE Antennas Propag. Mag..

[3] Ponsford A.M. (2001). Surveillance of the 200 nautical mile Exclusive Economic Zone (EEZ) using high frequency surface wave radar. Can. J. Remote Sens..

[4] FM/CW Radar Signals and Digital Processing. Technical Report. https://repository.library.noaa.gov/view/noaa/18645.

[5] Jankiraman M. (2018). FMCW Radar Design.

[6] Dzvonkovskaya A., Gurgel K., Rohling H., Schlick T. Low Power High Frequency Surface Wave RadarApplication for Ship Detection and Tracking. Proceedings of the International Conference on Radar.

[7] Gurgel K., Schlick T. Remarks on Signal Processing in HF Radars Using FMCW Modulation. Proceedings of the International Radar Symposium IRS 2009.

[8] The Effects of Sea Clutter on the Performance of HF Surface Wave Radar in Ship Detection and the Implication on the Radar Design. Defence Research and Development Canada–Ottawa Technical Memorandum DRDC Ottawa TM 2007-325. https://www.researchgate.net/publication/317290451.

[9] Menelle M., Auffray G., Jangal F., Bazin V., Urbani B. HF-Surface Wave Radar: First results for sea state studies. Proceedings of the 7th WSEAS International Conference on Application of Electrical Engineering (AEE’08).

[10] Ponsford A.M., Wang J. (2010). A review of high frequency surface wave radar for detection and tracking of ships. Turk. J. Electr. Eng. Comput. Sci..

[11] Gurgel K.W., Essen H.H., Kingsley S.P. (1999). HF radars: Physical limitations and recent developments. Coast. Eng..

[12] Headrick J.M., Thomason J.F. (1998). HF radars: Applications of high-frequency radar. Radio Sci..

[13] Sevgi L., Ponsford A., Chan H.C. (2001). Integrated Maritime surveillance system based on high–frequency surface–wave radars. 1. Theoretical background and numerical simulations. IEEE Antennas Propag. Mag..

[14] Ponsford A., Sevgi L., Chan H.C. (2001). An integrated Maritime surveillance system based on high–frequency surface–wave radars. 2. Operational status and system performance. IEEE Antennas Propag. Mag..

[15] Menelle M., Auffray G., Jangal F. Full Digital High Frequency Surface Wave Radar: French Trials in the Biscay Bay. Proceedings of the 2008 International Conference on Radar.

[16] Dzvonkovskaya A., Gurgel K., Rohling H., Schlick T. (2009). HF Radar WERA Application for Ship Detection and Tracking. J. Navig..

[17] Rohling H. (1983). Radar cfar thresholding in clutter and multiple target situations. IEEE Trans. Aerosp. Electron. Syst..

[18] Turley M. Hybrid CFAR techniques for HF radar. Proceedings of the Radar Systems (Conf. Publ. No. 449).

[19] Dzvonkovskaya A.L., Rohling H. Adaptive thresholding for HF radar ship detection. Proceedings of the Sixth International Radiowave Oceanography Workshop (ROW2006).

[20] Hongbo L., Yiying S., Yongtan L. (2007). Estimation of detection threshold in multiple ship target situations with HF ground wave radar. J. Syst. Eng. Electron..

[21] Jangal F., Saillant S., Helier M. (2008). Wavelet contribution to remote sensing of the sea and target detection for a high-frequency surface wave radar. IEEE Geosci. Remote Sens. Lett..

[22] Li Q., Zhang W., Li M., Niu J., Jonathan Wu Q.M. (2017). Automatic detection of ship targets based on wavelet transform for HF surface wavelet radar. IEEE Geosci. Remote Sens. Lett..

[23] Grosdidier S., Baussard A. (2012). Ship detection based on morphological component analysis of high-frequency surface wave radar images. IET Radar Sonar Navig..

[24] Starck J.L., Elad M., Donoho D. (2004). Redundant multiscale transforms and their application for morphological component separation. Adv. Imag. Electron Phys..

[25] Starck J.L., Moudden Y., Bobin J., Elad M., Donoho D. Morphological component analysis. Proceedings of the SPIE Conference Wavelets.

[26] Cai J., Zhou H., Huang W., Wen B. (2021). Ship Detection and Direction Finding Based on Time-Frequency Analysis for Compact HF Radar. IEEE Geosci. Remote Sens. Lett..

[27] Fast 2D Peak Finder. https://github.com/adinatan/fastpeakfind/releases/tag/1.13.0.0.

[28] Schmidt R. (1986). Multiple emitter location and signal parameter estimation. IEEE Trans. Antennas Propag..

[29] Capon J. (1969). High resolution frequency-wavenumber spectrum analysis. Proc. IEEE.

[30] Roy R., Kailath T. (1989). ESPRIT-estimation of signal parameters via rotational invariance techniques. IEEE Trans. Acoust. Speech Signal Process..

[31] Zhang L., Shi C., Niu J., Ji Y., Jonathan Wu Q.M. (2022). DOA Estimation for HFSWR Target Based on PSO-ELM. IEEE Geosci. Remote Sens. Lett..

[32] Kim B., Jin Y., Lee J., Kim S. (2021). High-Efficiency Super-Resolution FMCW Radar Algorithm Based on FFT Estimation. Sensors.

[33] Kim B., Jin Y., Lee J., Kim S. (2022). FMCW Radar Estimation Algorithm with High Resolution and Low Complexity Based on Reduced Search Area. Sensors.

[34] Li Y.-C., Choi B., Chong J.-W., Oh D. (2018). 3D Target Localization of Modified 3D MUSIC for a Triple-Channel K-Band Radar. Sensors.

[35] Seo J., Lee J., Park J., Kim H., You S. (2021). Distributed Two-Dimensional MUSIC for Joint Range and Angle Estimation with Distributed FMCW MIMO Radars. Sensors.

[36] Patole S., Torlak M., Wang D., Ali M. (2017). Automotive radars: A review of signal processing techniques. IEEE Signal Process. Mag..

[37] Kim B., Kim S., Lee J. (2018). A novel DFT-based DOA estimation by a virtual array extension using simple multiplications for FMCW radar. Sensors.

[38] Kim B., Kim S., Jin Y., Lee J. (2020). Low-complexity joint range and Doppler FMCW radar algorithm based on number of targets. Sensors.

[39] Akaike H. (1969). Fitting autoregressive models for prediction. Ann. Inst. Stat. Math..

[40] Rissanen J. (1978). Modeling by shortest data description. Automatica.

[41] Wax M., Ziskind I. (1989). Detection of the Number of Coherent Signals by the MDL Principle. IEEE Trans. Acoust. Speech. Signal Process..

[42] Ji Y., Zhang J., Wang Y., Chang G., Sun W. Performance Analysis of Target Detection with Compact HFSWR. Proceedings of the 2016 CIE International Conference on Radar (RADAR).

[43] Ji Y., Zhang J., Wang Y., Sun W., Li M. (2018). Target Monitoring Using Small-Aperture Compact High-Frequency Surface Wave Radar. IEEE A E Syst. Mag..

[44] Xie J., Yuan Y., Liu Y. (1998). Super-Resolution Processing for HF Surface Wave Radar Based on Pre-Whitened MUSIC. IEEE J. Ocean. Eng..

[45] MoyWe D.E., Warrington E.M. Some super-resolution DF measurements within the HF band. Proceedings of the 10th International Conference on Antennas and Propagation.

[46] Akaike H. (1974). A New Look at the Statistical Model Identification. IEEE Trans. Automat. Contr..

